# Challenging the Roles of NSP3 and Untranslated Regions in Rotavirus mRNA Translation

**DOI:** 10.1371/journal.pone.0145998

**Published:** 2016-01-04

**Authors:** Matthieu Gratia, Patrice Vende, Annie Charpilienne, Hilma Carolina Baron, Cécile Laroche, Emeline Sarot, Stéphane Pyronnet, Mariela Duarte, Didier Poncet

**Affiliations:** 1 Institut de Biologie Integrative de la Cellule (I2BC), UMR 9198, Département de Virologie, USC INRA 1358, Gif sur Yvette, France; 2 Université d’Evry Val d’Essonne, Département de Biologie, Evry, France; 3 INSERM UMR-1037 - Université de Toulouse III-Paul Sabatier, Laboratoire d'Excellence Toulouse Cancer (TOUCAN), Equipe labellisée Ligue Contre le Cancer Toulouse, France; University of British Columbia, CANADA

## Abstract

Rotavirus NSP3 is a translational surrogate of the PABP-poly(A) complex for rotavirus mRNAs. To further explore the effects of NSP3 and untranslated regions (UTRs) on rotavirus mRNAs translation, we used a quantitative in vivo assay with simultaneous cytoplasmic NSP3 expression (wild-type or deletion mutant) and electroporated rotavirus-like and standard synthetic mRNAs. This assay shows that the last four GACC nucleotides of viral mRNA are essential for efficient translation and that both the NSP3 eIF4G- and RNA-binding domains are required. We also show efficient translation of rotavirus-like mRNAs even with a 5’UTR as short as 5 nucleotides, while more than eleven nucleotides are required for the 3’UTR. Despite the weak requirement for a long 5’UTR, a good AUG environment remains a requirement for rotavirus mRNAs translation.

## Introduction

Eukaryotic mRNA translation initiation occurs for the vast majority of cellular mRNAs by scanning of the 5' untranslated region (UTR) by the small ribosomal subunit in complex with the initiator methionine-tRNA and additional translation initiation factors [1, 2] until it recognizes the AUG start codon. Start codon recognition triggers joining of the large (60S) ribosomal subunit, which forms a complete 80S ribosome.

Scholars believe that eIF4E recognition of the mRNA 5' cap structure is the first step toward mRNA loading on the ribosomal small subunit. The presence of a 5' cap enhances translation of mRNA 3- to 30-fold [3]. Additional characteristics of the 5'UTR and AUG start codon environment necessary for efficient translation initiation have also been identified. The best-characterized AUG context for vertebrate mRNAs is RCCAUGG (R for purine nucleotide), which is better known as the "Kozak sequence" [4]. The R and G at positions -3 and +4 relative to the A of the AUG have been described the most important positions. When both the -3 and +4 positions match the Kozak sequence, the AUG context is considered "strong" and yields efficient initiation at this start codon. Initiation is weaker when only one of the two positions matches the Kozak sequence and even poorer when none of the two positions matches the Kozak sequence. The -3 and +4 positions stabilize the PIC at the start codon, and a weak context allows PIC read-through and initiation at a downstream AUG (leaky scanning).

The 5’UTR length is also important [5]. At least 20 nt are required to efficiently recognize AUG in a strong context. Conversely, a 5’UTR less than 20 nt-long yields a high proportion of leaky scanning. This minimal length requirement likely results from ribosomal protection 15 nt upstream of the AUG and PIC contacts at 10 to 20 more nucleotides on the 5' side [6–8].

Certain mRNA molecules are translated by mechanisms that do not conform to this conventional scanning-initiation model but instead use a specific mechanism. Well characterized examples include the so-called internal ribosome entry sites (IRES) [9, 10], TISU (translation initiator of short 5’UTR) [11], or stem-loop-mediated initiation of certain histone mRNAs [12].

The 3'UTRs bear specific, known sequences involved in controlling protein expression [13]; however, the stop codon environment and 3'UTR length requirements for efficient translation are less well-known. The base immediately after the stop codon modulates termination efficiency; a purine is more efficient than C or U [14], and short 3’UTRs repress translation [15].

Group A Rotavirus (RVA) mRNA molecules are capped and bear short 5' and 3’UTRs. As exemplified by the RF rotavirus strain, 5’UTRs (average* 30 ±8) vary from 9 (gene 4) to 50 nt (gene 3), and the 3'UTRs (average* 67 ±30) vary from 17 (gene 1) to 182 (gene 10) (*not considering the gene 11 ORF2 that encodes NSP6, which is not expressed by all viruses). The RVA 5’UTRs begin with a conserved GGCU sequence and 3’UTRs end with the conserved GACC sequence. Remarkably, rotavirus mRNAs are not polyadenylated; however, they are well-translated during infection.

The viral non-structural protein NSP3 is bound to the 3’ end of the viral mRNA during infection [16], and NSP3 dimers can specifically bind the terminal GACC sequence [17, 18]. NSP3 dimers also bind to the eIF4G translation initiation factor [19, 20]. The simultaneous interaction between NSP3 and the viral mRNA 3’ end and the eIF4G enhances translation of rotavirus-like reporter mRNA [21–23] just as PABP interaction with polyadenylated mRNA and eIF4G.

The NSP3-stimulated translation requirement for viral protein production in cell-culture-adapted rotavirus strains that grow on poorly differentiated epithelial cells (MA104) has been questioned [24]. Through siRNA-mediated down-regulation of NSP3 in infected cells, Montero et al. did not observed an overall decrease in viral protein production via pulse labeling, although a transient decrease was visible early after infection. The authors proposed a model in which the NSP3 RNA- and eIF4G-binding domains function independently, the RNA-binding domain (RNA-BD) enhances translation solely by protecting RNA from degradation, and the eIF4G-binding domain (4G-BD) down regulates cellular mRNA translation. We recently established that apart from viral infection, NSP3 expression enhances rotavirus-like mRNA translation hundreds fold without protecting the mRNA and does not inhibit polyadenylated mRNA translation but, conversely, moderately stimulates translation [23].

To decipher the roles of the rotavirus mRNA 5' and 3’UTRs and the NSP3 domains in NSP3-dependent translation, we first constructed an in vivo assay using electroporation of in vitro-synthesized reporter mRNA in cells transiently expressing NSP3. This assay was then used to define the minimal 5’ and 3’UTRs required for efficient translation of rotavirus-like mRNA and to address the dependence of RVA mRNA translation on the "Kozak" environment.

## Results

### I- Development of a quantitative in vivo assay to study the roles of NSP3 and RNA sequences on rotavirus-like mRNA translation efficiency

**Fig 1:**
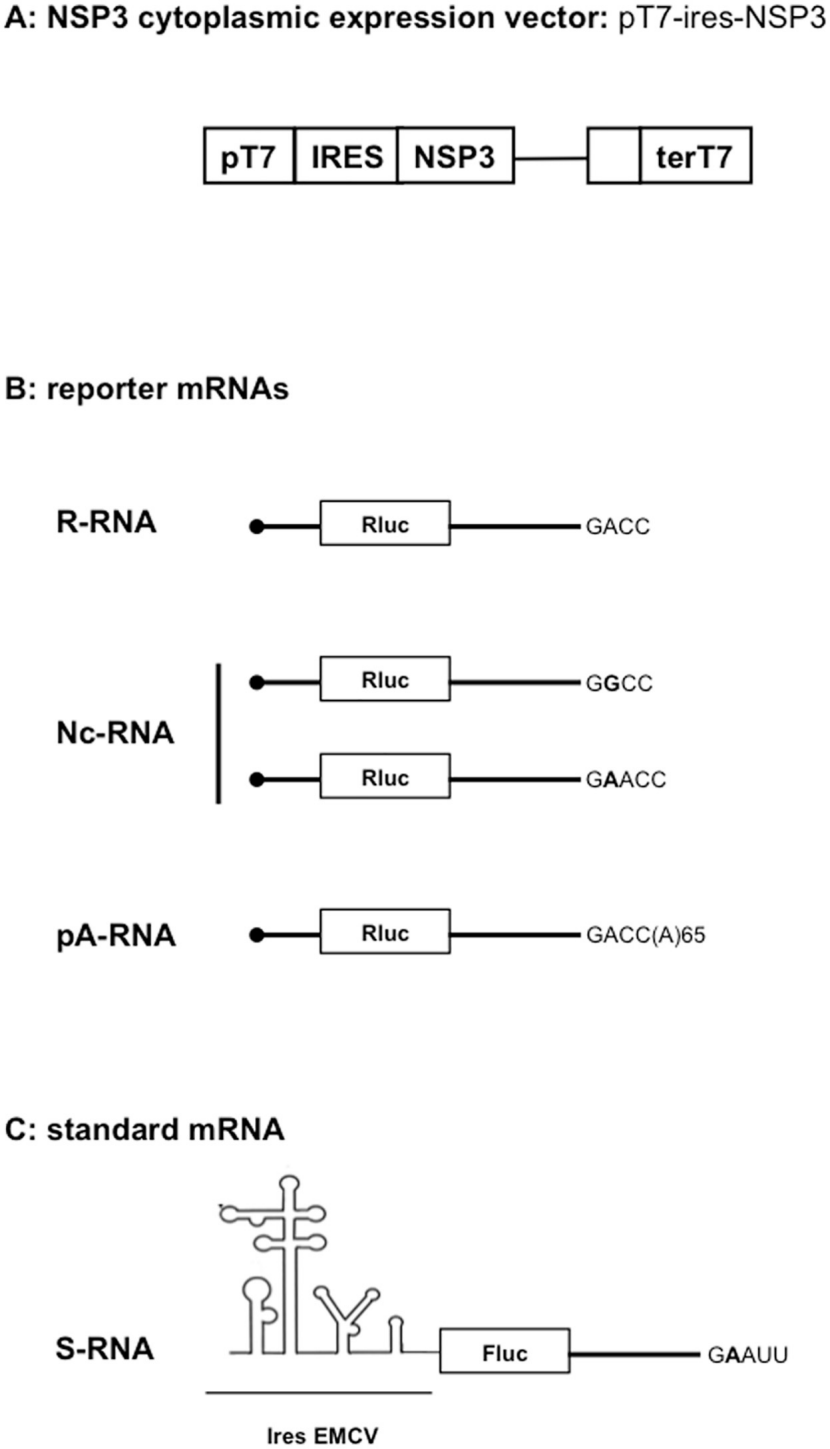
Schematic representation of the NSP3 cytoplasmic-expression vector (A), reporter mRNA (B) and standard mRNA (C) used. The NSP3 expression vector was transfected as DNA in BSRT7 cells. The capped reporter mRNA ending with the canonical GACC (R-RNA) or non-canonical (Nc-RNA) terminal sequence was electroporated with the standard RNA (S-RNA).

Our assay uses three types of plasmid vectors (S1 Fig). 1-The expression vectors (Fig 1A) facilitate cytoplasmic expression of NSP3 (or eGFP or mutated NSP3) after plasmid DNA transfection into cells expressing T7 RNA polymerase. 2- The reporter vectors are used as templates for in vitro synthesis of i- rotavirus-like reporter mRNAs (Fig 1B) bearing a luciferase ORF inserted between rotavirus gene 5' and 3’UTRs and with a canonical (R-RNA) or noncanonical (Nc-RNA) 3' terminal sequence or ii- cellular-like poly-adenylated mRNAs (pA-RNA, Fig 1B) bearing the same ORF and UTRs as the R-RNA but extended by 65 adenines at the 3' end. 3- The standard vector (Fig 1C) is used as template for in vitro synthesis of an mRNA (S-RNA), the translation of which is not influenced by NSP3 and which facilitates reporter mRNA transfection standardization. Transfection of the NSP3-expression vector (as DNA) followed by co-electroporation of the in vitro synthesized reporter and standard mRNAs facilitates quantitative measurements of NSP3’s (or its mutants’) effects on reporter mRNA translation and a comparison of reporter mRNA molecules with different 5' or 3’UTRs. A more detailed description of the assay is provided in S1 and S2 Figs and S1 Text.

### II- The importance of the cap structure in NSP3-dependent translation

**Fig 2:**
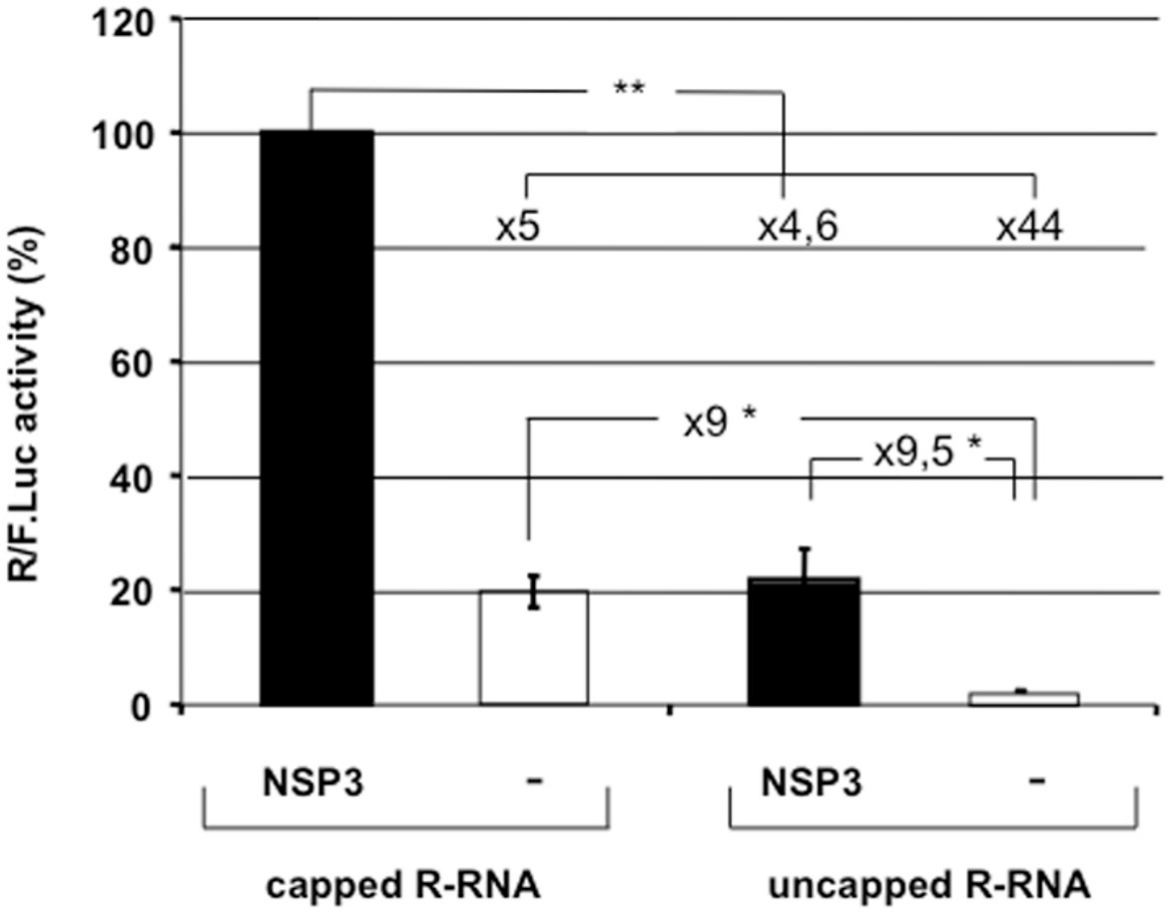
The importance of the 5' cap and NSP3 expression for rotavirus-like reporter mRNA translation. BSRT7 cells expressing NSP3 or eGFP(-) were electroporated with capped (left) or uncapped (right) reporter rotavirus-like mRNA molecules and the standard mRNA. *Renilla* and firefly luciferase activities were measured 6 hours after electroporation. The *Renilla* to firefly luciferase ratio is relative to the control, which was considered 100. The data are presented as the mean ± standard error of the mean (SEM) of three independent experiments in triplicate. The fold increases are indicated and a two-tailed Student’s *t*-test was used *p<0.05; **p<0,01).

To quantify the relative contributions of either the 5’ cap or NSP3 on rotavirus-like mRNA translation, capped and uncapped R-RNA were synthesized and used in our assay in the presence or absence of NSP3. Fig 2 illustrates the results for the two types of RNA with or without NSP3. Capped mRNA translation in cells expressing NSP3 (Fig 2) was stimulated 44-fold relative to the uncapped mRNA in cells expressing eGFP. The relative contributions of either the 5' cap or NSP3 to the stimulated mRNA translation were similar (approximately 9-fold compared to the translation of uncapped mRNA in absence of NSP3 (Fig 2)). Moreover, the weak activities of the uncapped reporter mRNA show that the small fraction of uncapped mRNAs that may be present in the capped mRNA preparations did not contribute significantly to the luciferase signal.

### III- The importance of the mRNA 3’ end GACC sequence in NSP3-dependent translation

**Fig 3:**
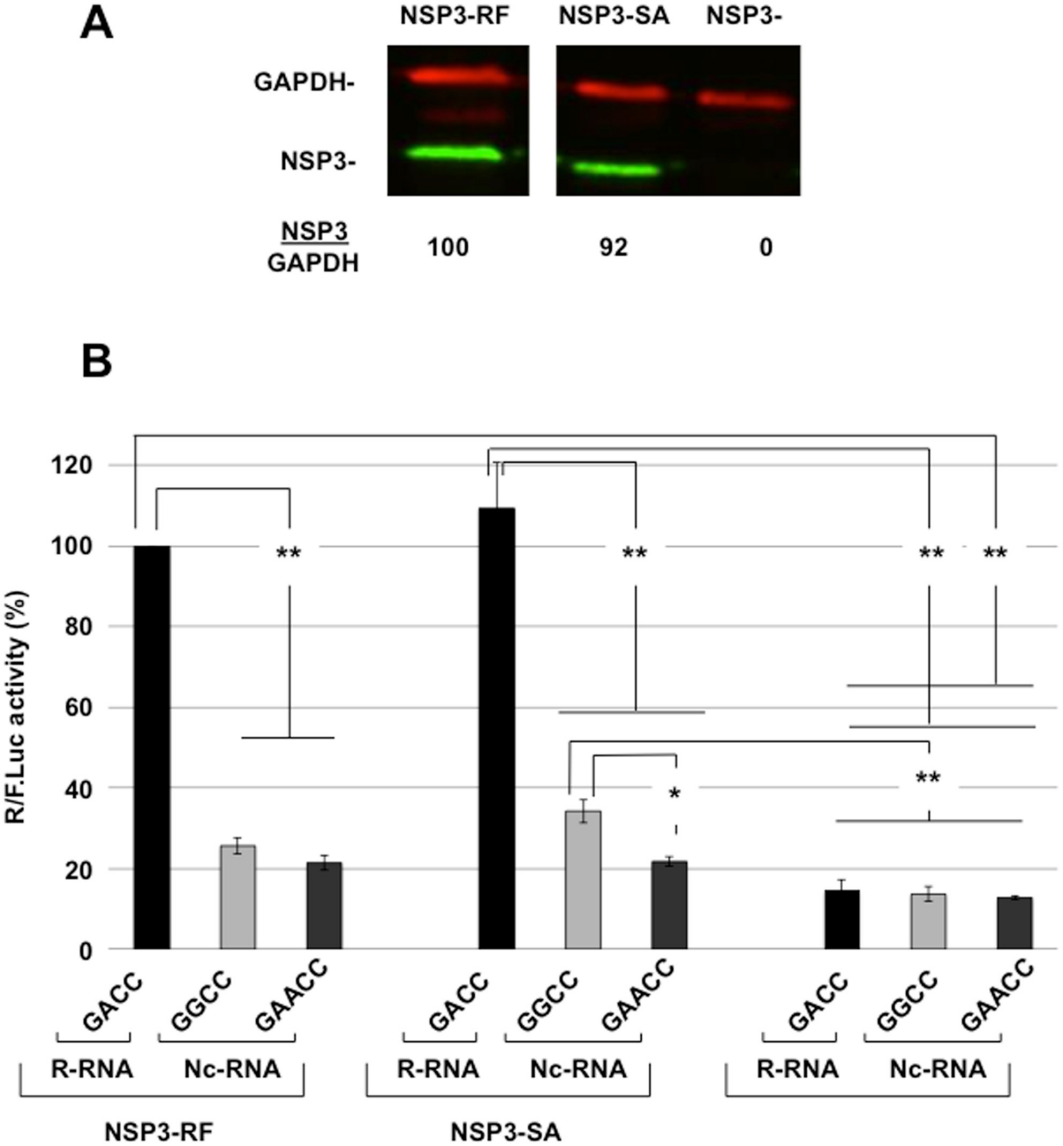
The importance of the 3' end and NSP3 origin for rotavirus-like reporter mRNA translation. **A:** Cytoplasmic expression of NSP3-RF and NSP3-SA in BSRT7 cells. Lysate from BSRT7 cells transfected for 24 h with expression vectors encoding NSP3 from the RF (NSP3-RF) or SA11 (NSP3-SA) RVA strains or no NSP3 (NSP3-KO). The lysates were analyzed (using the same western blot but NSP3-RF and NSP3-SA samples were not side-by-side) with an anti-NSP3 rabbit polyclonal antibody and a mouse monoclonal antibody against the cellular protein GAPDH (used as a loading control). The ratio of NSP3 versus GAPDH fluorescence (NSP3/GAPDH) is indicated at the bottom of the figure. **B:** BSRT7 cells expressing NSP3 from the RF (NSP3-RF) or SA11 (NSP3-SA) RVA strains or no NSP3 were electroporated with the indicated capped reporter mRNA (R- or Nc-RNA) and the standard RNA. The *Renilla* and firefly luciferase activities were measured 6 hours after electroporation. The *Renilla* to firefly luciferase ratio is relative to the control (R-RNA in cells expressing NSP3-RF), which was considered 100. The data are the mean ± SEM of three independent experiments in triplicate. A two-tailed Student’s *t*-test was used (*p<0.05,**p<0.01)

The 3' end differs from the 3’ GACC canonical sequence in a few rotavirus genes. In the SA11-4F strain, the 3’ end of gene 7 (encoding NSP3) is GGCC and that of gene 5 (encoding NSP1) is GAACC whereas gene 5 from the RRV strain ends with GGCC [25]. These non-canonical (Nc) sequences are not recognized by NSP3 from the RF (NSP3-RF) [18] or RRV strains [25]. To test whether NSP3 from SA11 (NSP3-SA) evolved to recognize these sequences, BSRT7 cells expressing NSP3-RF or NSP3-SA at similar levels (Fig 3A) were electroporated with R-RNA or Nc-RNAs ending with -GGCC or -GAACC. The results in Fig 3B show that translation of the reporter RNAs with Nc 3’ end sequences decreased by 3- to 5-fold compared with the canonical R-RNA; however, we did not observed a difference between either reporters introduced into BSRT7 cells that did not express NSP3. A slight but significant increase in Nc-RNA-GGCC translation was observed with NSP3-SA, which might indicate that NSP3-SA recognized the GGCC sequence slightly better than NSP3-RF. These results clearly show that NSP3 and the GACC sequence are both required for efficient rotavirus-like mRNA translation and that our assay detects slight differences in stimulated translation by NSP3 proteins from different origins.

### IV- The importance of the NSP3 eIF4G- and RNA-binding domains

**Fig 4:**
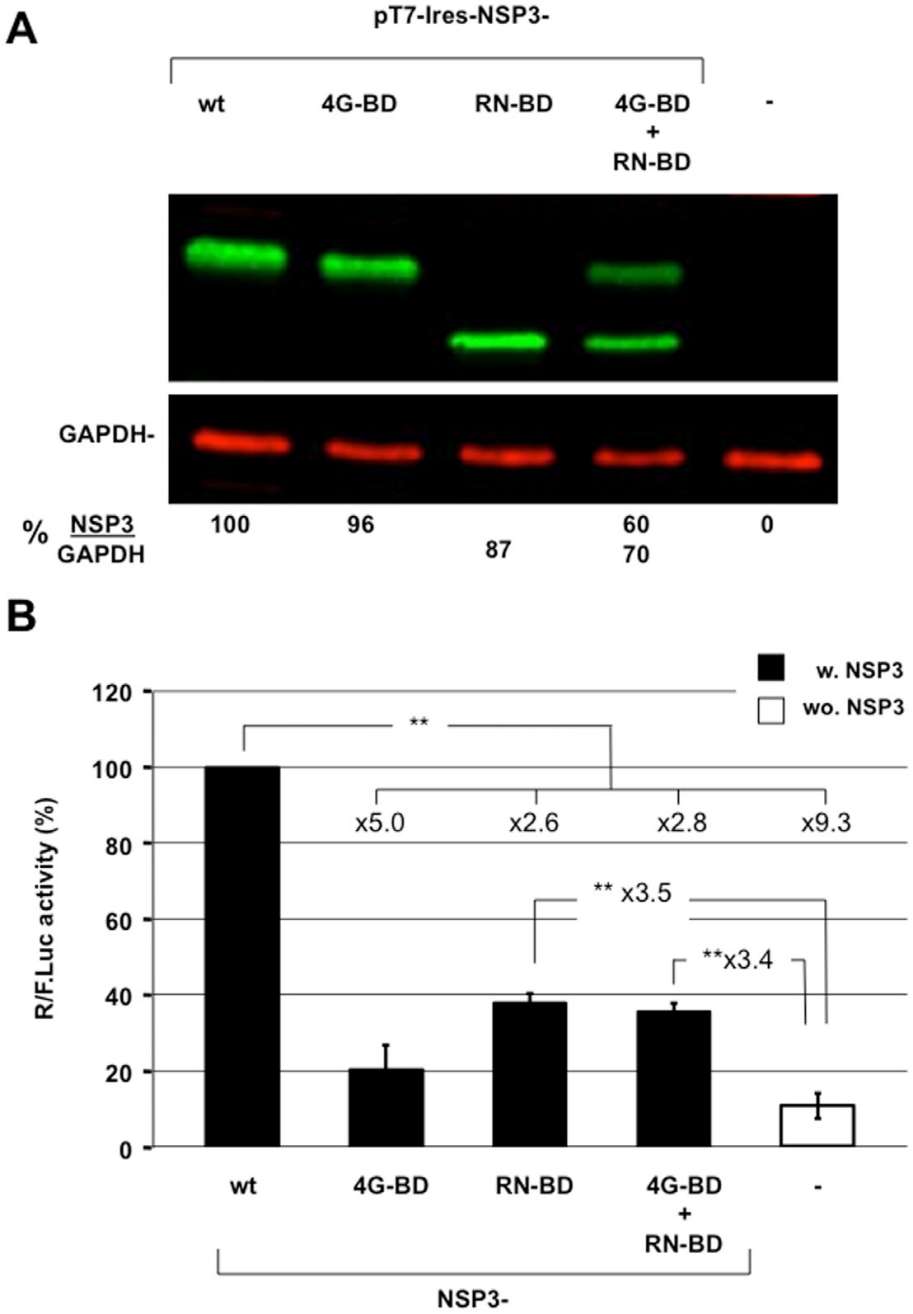
The importance of the NSP3 eIF4G- and RNA-binding domains. **A:** Expression of wild type or mutated NSP3. Lysate from BSRT7 cells transfected for 24 h with the expression vectors encoding wild type NSP3 (wt); NSP3 mutated at the RNA-binding domain (4G-BD) or the eIF4G-binding domain (RN-BD) alone or in combination; or no NSP3 (NSP3-KO) was analyzed using western blotting with an anti-NSP3 rabbit polyclonal antibody and a mouse monoclonal antibody against the cellular protein GAPDH (used as a loading control). The ratio of NSP3 versus GAPDH fluorescence (NSP3/GAPDH) is indicated at the bottom of the figure. **B:** BSRT7 cells expressing wild type or mutated NSP3 were electroporated with the capped R-RNA and standard RNA. The *Renilla* and firefly luciferase activities were measured 6 hours after electroporation. The *Renilla* to firefly luciferase ratio is relative to the control (R-RNA in cells expressing NSP3-RF), which was considered 100. The data are the mean ± SEM of three independent experiments in triplicate. The fold increases are indicated and a two-tailed Student’s *t*-test was used (*p<0.05; **p<0.01).

It has been suggested that NSP3-dependent translation of viral mRNA is solely due to viral mRNA 3' end protection by NSP3 binding and that an interaction between NSP3 and eIF4G is not required [24]. To test for the role of the different NSP3 domains in translation, the RNA-binding domain or the 4G- binding domain were expressed either separately or together by co-transfection in BSRT7 cells (Fig 4A). The RNA- binding domain corresponding to amino-acid position 3 to 249 of NSP3. The 4G- binding domain corresponding to full-length NSP3 with mutations at positions R83 and N84 to alanines. Expression of the reporter R-RNA was compared with the negative and positive controls, which were generated through expressing the same reporter mRNA in cells expressing eGFP or wild-type NSP3, respectively. Each NSP3 protein was expressed at comparable levels (Fig 4A), but the RN-BD or 4G-BD expression was half because the total quantity of co-transfected DNAs remained constant. As illustrated by Fig 4B, wild type NSP3 expression enhanced rotavirus-like reporter translation >9-fold compared with translation in cells expressing eGFP. Expression of each NSP3 binding domain alone failed to enhance translation as efficiently as the full-length NSP3, and co-expression of the binding domains did not yield better translation. Potential protection of the viral-like mRNA by the NSP3 RNA-BD accounted for less than half of the full-length NSP3 expression. Moreover, this expression did not differ significantly with expression of 4G-BD alone. Thus, the two NSP3 domains do not operate independently.

### V- Positive feedback for NSP3 mRNA translation

**Fig 5:**
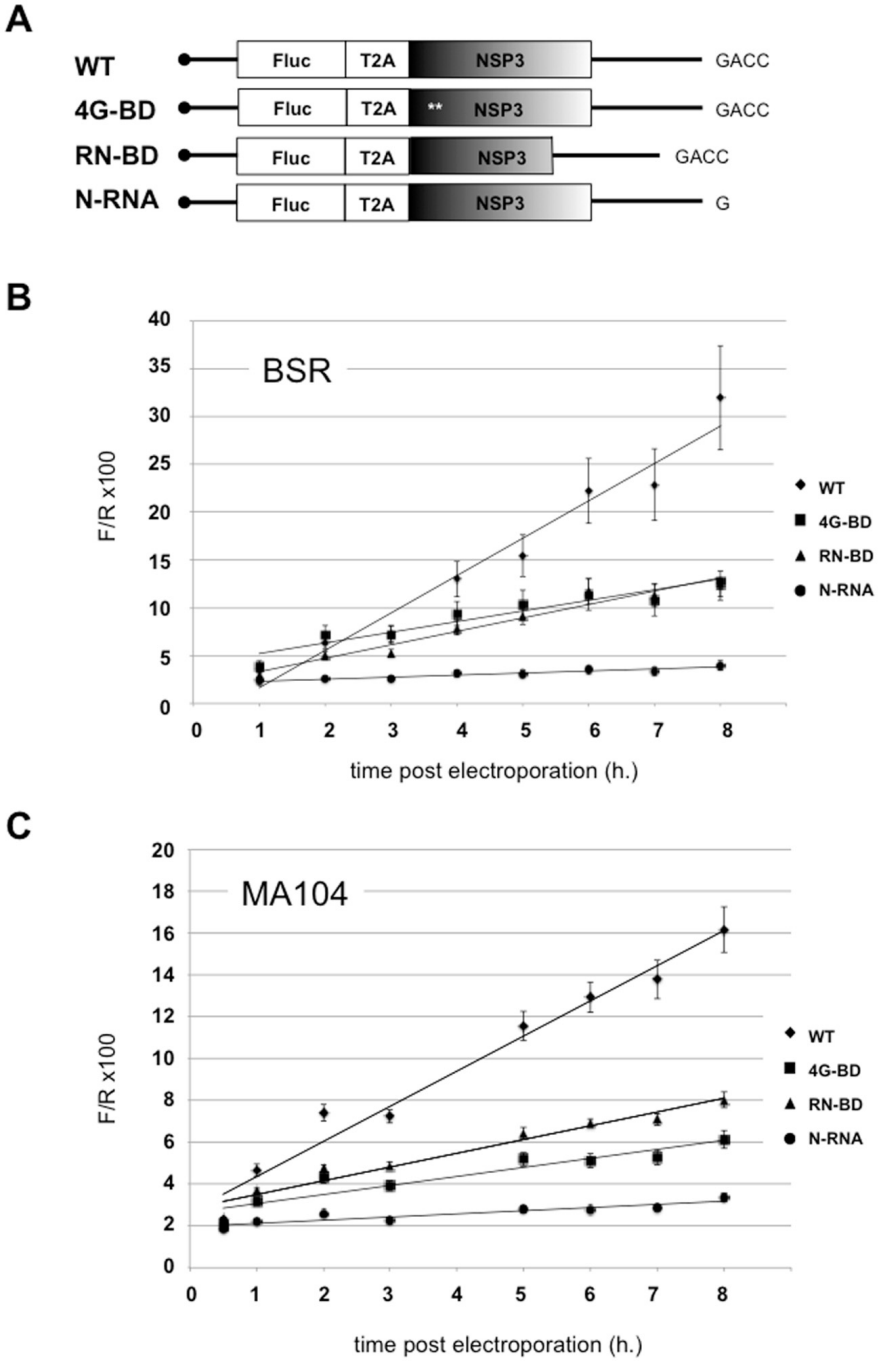
Positive feedback for translation of rotavirus mRNA encoding NSP3. **A:** schematic representation of the "T2A reporter" mRNA encoding firefly luciferase and wild type or mutated NSP3 ending or not with the GACC sequence. **B**: BSRT7 cells or C; MA104 cells were electroporated with the capped "T2A reporter" mRNA and a standard *Renilla* RNA. The *Renilla* and firefly luciferase activities were measured from .5 to 8 hours after electroporation. The firefly to *Renilla* (F/R) luciferase ratio is reported. The data are the mean ± SEM of five independent experiments.

If NSP3 is required to translate rotavirus mRNA, how can NSP3-encoding mRNA be translated at the onset of infection when NSP3 is not present? To answer this frequently asked question, we constructed a self-stimulating reporter mRNA (Fig 5A). In this reporter mRNA, the firefly luciferase ORF is joined in frame with the NSP3 ORF using a "self-cleaving" peptide (T2A) from the *Thosea asigna* virus [26, 27]. Translation of the chimeric ORF produces firefly luciferase (with most of the T2A peptide fused to the C-terminus) and NSP3 with only one additional proline at the N-terminus. Because the mRNA encoding this ORF ends with GACC, the wild type NSP3 will recognize the 3' end of its mRNA, stimulate its translation and increase firefly luciferase expression (notably, with these constructs, the 3' and 5’UTRs are from the RF gene 7 encoding NSP3). Transfection of this "T2A reporter" mRNA in cells not expressing NSP3 mimics the first step of rotavirus infection; NSP3-stimulated translation is demonstrated through enhanced Firefly activity. Controls were provided by constructs with mutations in the NSP3 RNA- or eIF4G- binding domains and an mRNA encoding a wt NSP3 but with the GACC sequence deleted at the 3' end (Fig 5A). The RNA molecules were electroporated in BSRT7 cells (Fig 5B) or MA104 (Fig 5C) with *Renilla* S-RNA, and the luciferase activities were measured each hour from .5 to 8 hours after electroporation. The results are similar to the BSR and MA104 cells, which show (Fig 5) a steady increase in FlucT2ANSP3wtGACC mRNA translation and a modest increase in FlucT2ANSP3wtnona mRNA translation (the F/R ratio is 6-fold higher between the two RNA molecules 8 hours after transfection). Translation of the mRNA molecules encoding mutated NSP3s was 40 to 50% lower than for the FlucT2ANSP3wtGACC mRNA. These results show positive feedback between NSP3 and its encoding mRNA, wherein the 3' GACC end and both NSP3 domains are required.

### VI- The effect of 5’ and 3’UTRs on rotavirus-like mRNA translation

The above-described results show that our assay is functional and that it recapitulates known properties of NSP3-dependent translation, which requires a cap, a 3' GACC sequence [21, 22] and expression of full-length NSP3 [21]. Therefore, this assay was used to explore certain properties of the rotavirus gene 5’ and 3’UTRs by changing their length, sequence and the initiation codon environment.

#### VI-1- Length requirement for the rotavirus-like reporter 5’UTR; the case of the rotavirus gene four 5'UTR

We questioned whether rotavirus gene four 5’UTR-mediated translation operates in accordance with the standard model of translation initiation based on an examination of the rotavirus gene 4 (encoding the spike protein VP4) 5’UTR. This UTR is only 9 nt-long (from the first G to the initiation codon AUG), whereas an initiating ribosome with the AUG codon in the P site protects 12 to 17 nt on the 5’ side [8], and translation initiates most efficiently when the AUG is spaced approximately 50 nt away from the cap structure [28].

**Fig 6:**
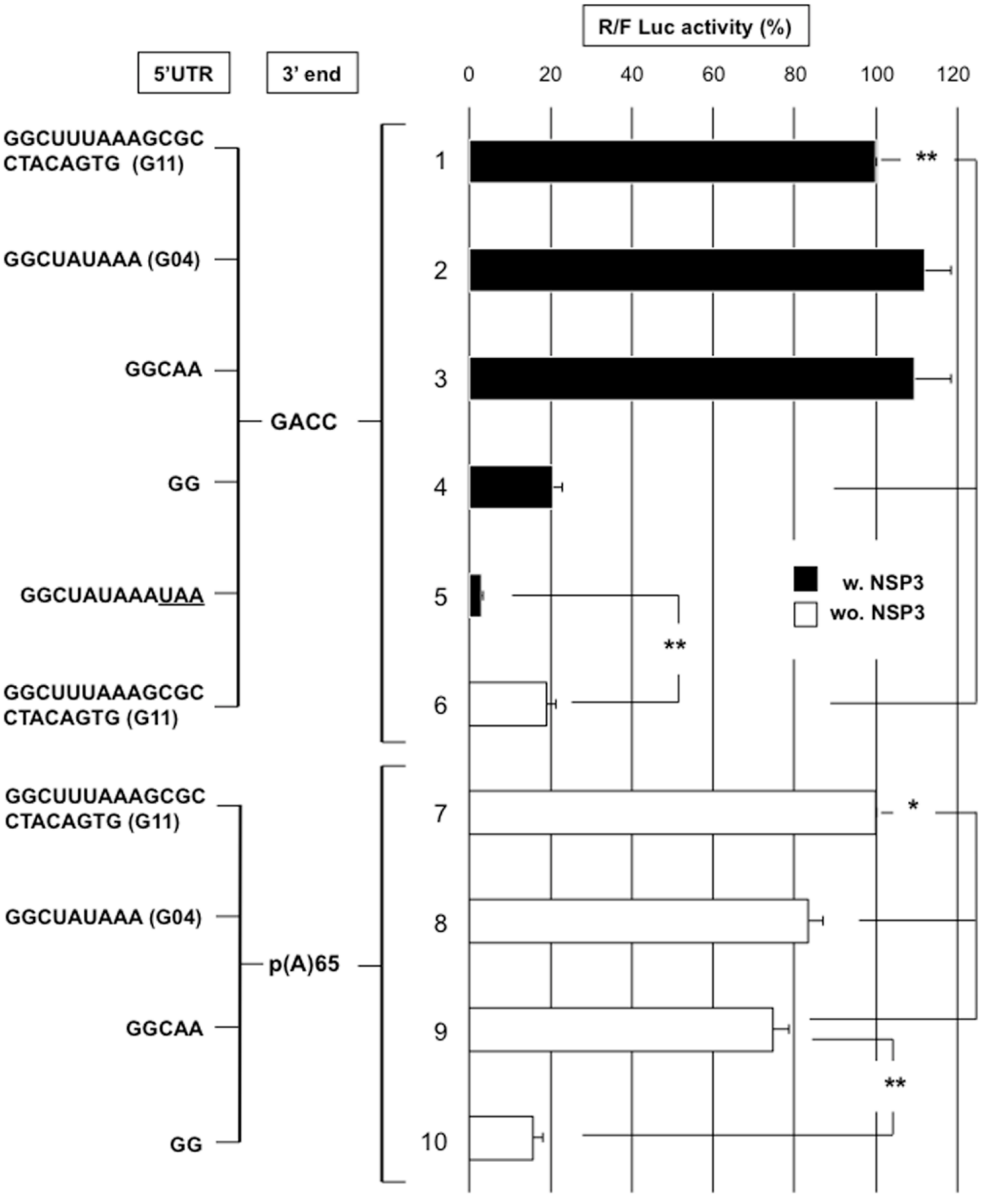
The importance of the 5'UTR length in NSP3- and poly(A)-dependent translation. BSRT7 cells expressing NSP3 (NSP3; black bars) or no NSP3 (NSP3-KO) (wo. NSP3; white bars) were electroporated with the indicated capped R-RNA molecule (lines 1–6) or capped -p(A)-RNA molecule (lines 7–10) and the standard RNA. The *Renilla* and firefly luciferase activities were measured 6 hours after electroporation. The *Renilla* to firefly luciferase ratio is relative to the controls (lines 1 and 7), which was considered 100. The nucleotide sequence upstream the AUG initiation codon is indicated and the 3’UTR is from gene 11. The data are the mean ± SEM of three independent experiments in triplicate. A Student’s *t*-test was used (*p<0.05;**p<0,01).

To test whether the 9 nt of gene 4 5'UTR facilitate conventional translation initiation, the full-length 5’UTR from the gene 4 RF was placed upstream of the Rluc ORF to produce the cap-RF04-RLuc-RF06-GACC reporter mRNA (Fig 6 line 2). A control RNA with a stop codon substituted for the initiation codon was also used. Changing the first initiation codon into a stop codon fully abolishes reporter expression (Fig 6, line 5), which shows that translation did not initiate on this codon and that initiation at any downstream AUG codon did not produce an active luciferase protein (the second AUG in the Rluc ORF is out of frame, and the third AUG is in a bad Kozak environment and encodes a luciferase protein with 13 amino acids truncated). Translation did not differ among the reporter mRNA molecules with a 21, 9 or 5 nt-long 5'UTR (Fig 6 lines 1 to 3). Reducing the 5’UTR length to 2 nt decreased expression of the reporter mRNA 5-fold (line 4) to the level of expression without NSP3 (line 6). To determine whether this weak 5’UTR length requirement is specific for NSP3-dependent translation, the same reporter mRNA molecules were generated with a 65 nt-long poly(A) tail and used in BSRT7 cells not expressing NSP3 (Fig 6 lines 7–10)). Poly(A)-dependent translation was only slightly more sensitive to the 5’UTR length; we observed approximately 25% less translation for the 9 and 5 nt-long UTRs (lines 7–9). Similar to the NSP3-dependent translation, the 2 nt-long 5’UTR reduced polyadenylated mRNA expression 5-fold (line 10). Thus, a short 5’UTR is not a handicap for either rotavirus gene four translation or poly(A) mRNA translation.

#### VI-2- The Kozak context requirement

The initiation codon context largely influences fidelity and efficiency in translation initiation. A weak AUG context promotes ribosomal leaky scanning and downstream initiation.

**Fig 7:**
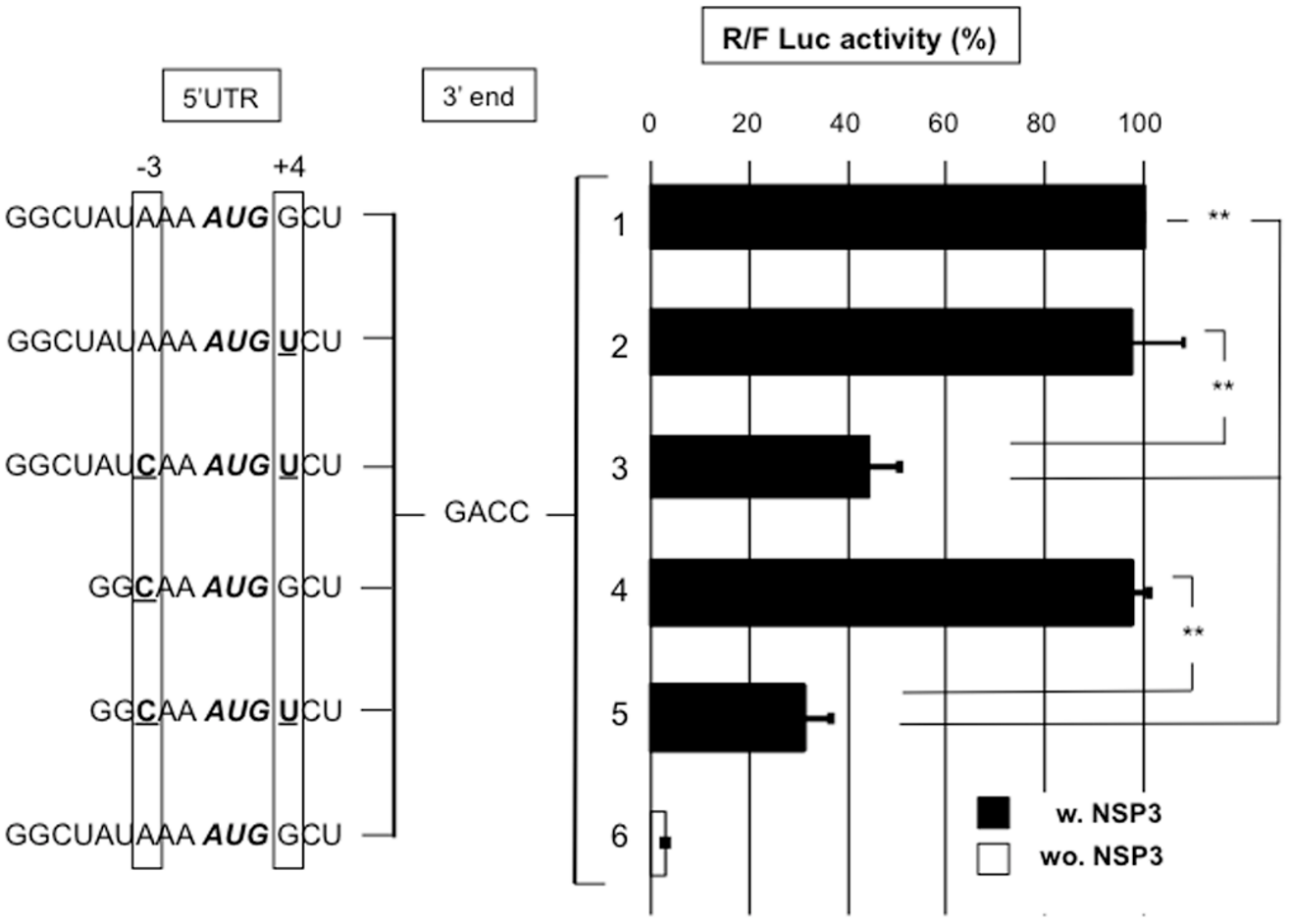
The importance of the initiation codon context in NSP3-dependent translation. BSRT7 cells expressing NSP3 (black bars) or no NSP3 (NSP3-KO) (wo. NSP3 white bar) were electroporated with the standard RNA and with capped R-RNA (lines 1–6) with 9- (lines 1–3,6) or 5- (lines 4,5) nt-long 5’UTRs with different AUG (bold and italicized) contexts. The -3 and +4 positions are boxed, and the nucleotides that differ from the wild type (line1) are underlined. The *Renilla* and firefly luciferase activities were measured 6 hours after electroporation. The ratio of *Renilla* to firefly luciferase activities is reported relative to the control (line 1), which was considered 100. The data are the mean ± SEM of three independent experiments in triplicate. A Student’s *t*-test was used (*p<0.05;**p<0,01).

The first initiation codon in gene 4 is in a good Kozak context (Fig 7 line 1); thus, we questioned whether the cap-RF04-RLuc-RF06-GACC mRNAs with a weak requirement for 5'UTR length requires a good Kozak context for efficient translation. Mutations were introduced at the –3 and/or +4 positions in the full-length or 5 nt-long gene four 5’UTR, and luciferase activity was measured after RNA electroporation. Fig 7 shows that a mutation at the +4 position on the 9 nt-long 5' UTR (lines 1 and 2) or at the -3 position on the 5 nt-long 5' UTR (line 4) did not affect translation; however, double mutations at +4 and -3 positions reduced translation 2-fold with both types of 5’UTR (lines 3 and 5). Thus, despite a short 5’UTR, which precludes the standard scanning mode in initiation, the cap-RF04-RLuc-RF06-GACC reporter mRNA translation is sensitive to the initiation codon context.

Interestingly, translation of the reporter mRNA with the gene four 5’UTR was more sensitive to the presence of NSP3 than the previously used reporter mRNA because translation decreased 25-fold without NSP3; however, reporters bearing the gene11 5'UTR typically yield a 5-fold decrease (Fig 7 lines 1 and 6).

#### VI-3- The 5' and 3’UTRs combined

The above results (Fig 6) show that whether the reporter mRNA is efficiently translated with the gene 4 5’UTR is likely not due to the RNA sequences in this UTR because a 5-nt 5’UTR stimulated translation as efficiently as the wild type 5’UTR. We questioned whether the presence of a translational enhancer in the gene six 3’UTR [29], which is in the reporter mRNA molecules used in Fig 6, accounts for these observations.

**Fig 8:**
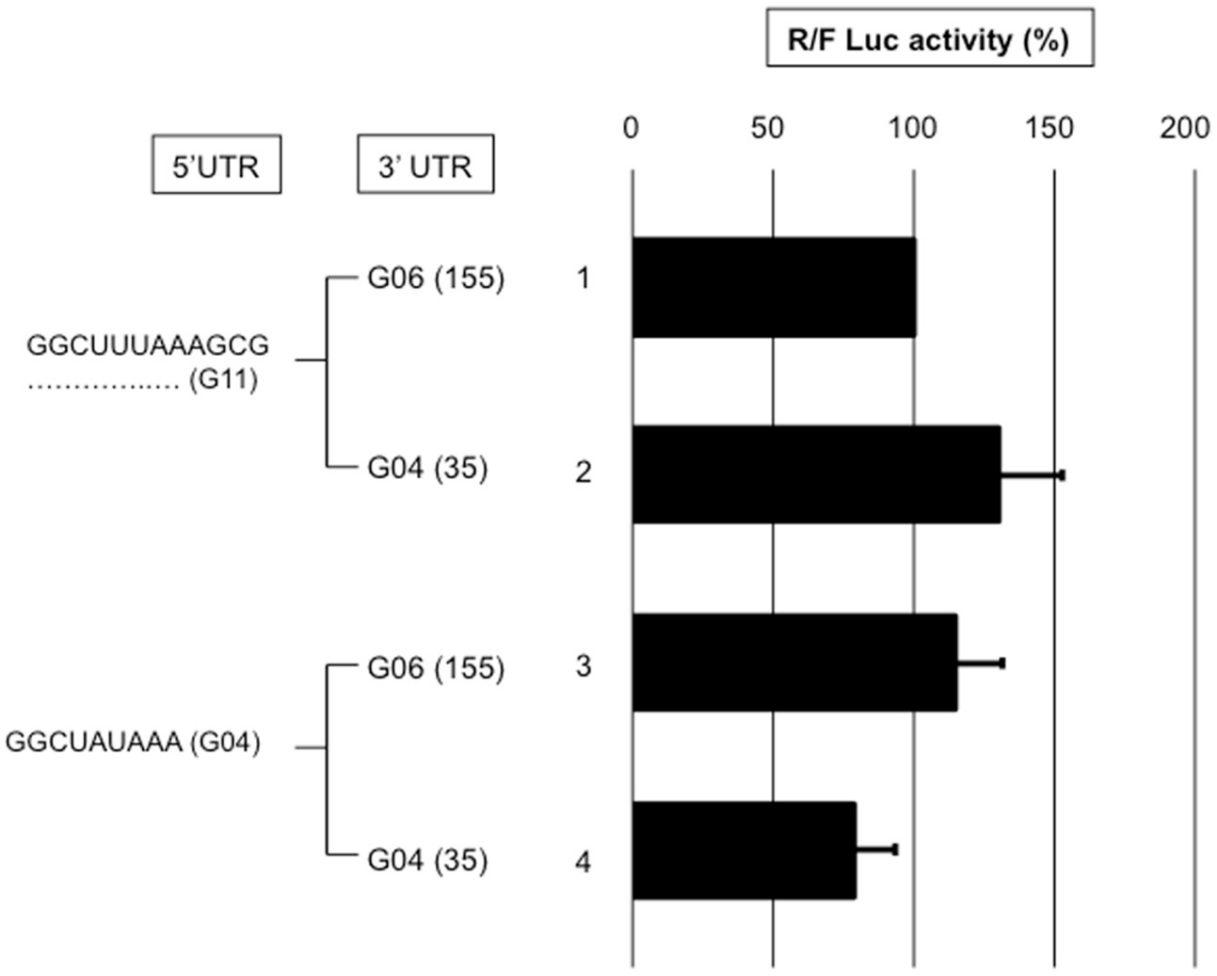
Comparison of 3'UTRs from genes 4 and 6 on NSP3-dependent translation efficiency. BSRT7 cells expressing NSP3 were electroporated with the standard RNA and capped R-RNA with the 5'UTR from gene 11 (lines 1,2) or 4 (lines3,4) combined with the 3’UTR from gene 6 (lines 1,3) or 4 (lines 2,4). The *Renilla* and firefly luciferase activities were measured 6 hours after electroporation. The ratio of *Renilla* to firefly luciferase activities is reported relative to the control (line 1), which was considered 100. The data are the mean ± SEM of three independent experiments in triplicate. A Student’s *t*-test was used (*p<0.05;**p<0,01).

**Fig 9:**
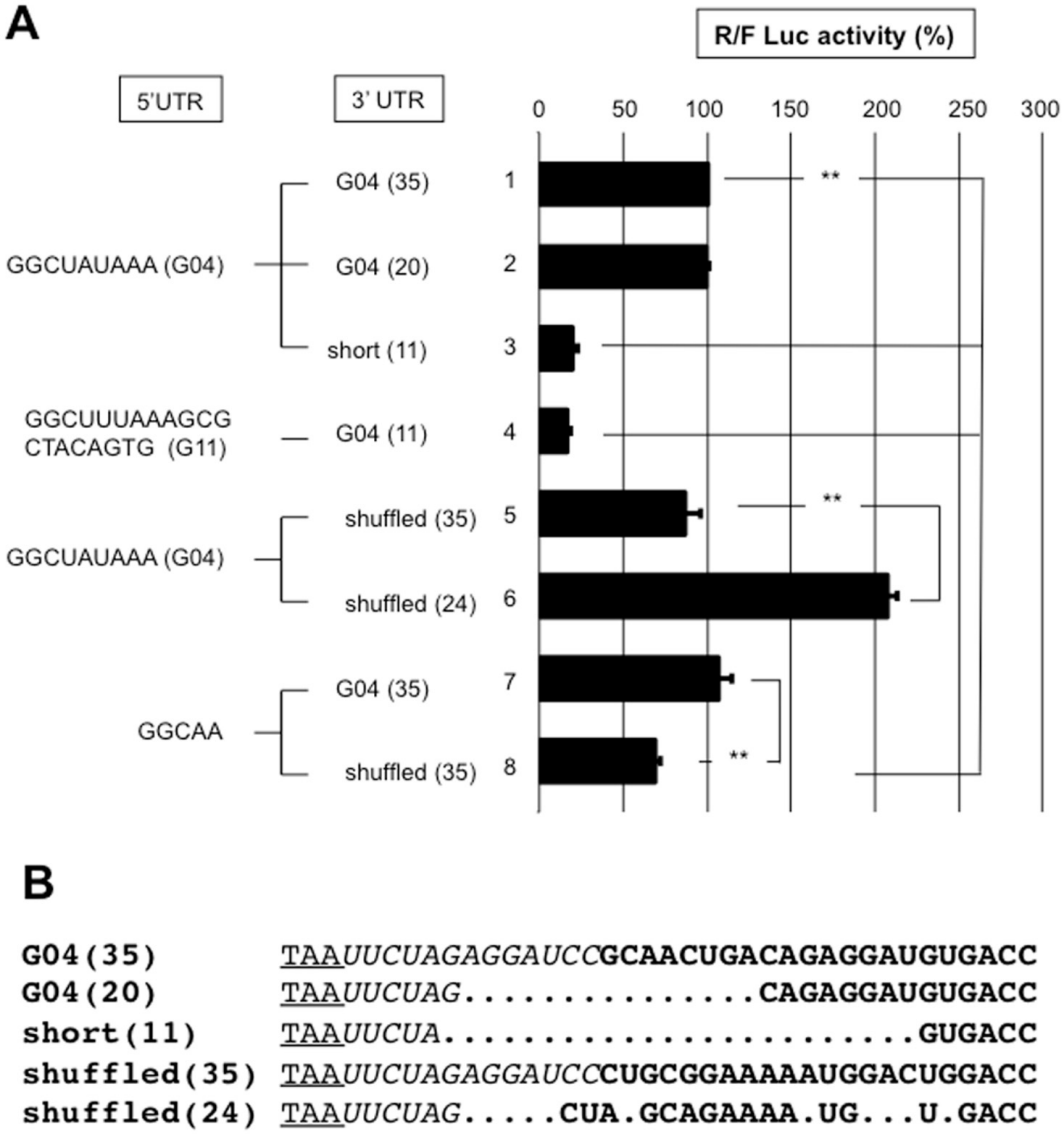
The roles of the 3'UTRs sequences on NSP3-dependent translation efficiency. **A**: BSRT7 cells expressing NSP3 were electroporated with the S-RNA and capped R-RNA with the 5'UTR from gene 4 (lines1-3,5,6), 11 (line 4) or a 5 nt-long UTR (lines 7,8) combined with the full-length 3’UTR from gene 4 wild-type (lines 1,4,7); shuffled (lines 5,8); shortened to 20 (line 2) or 11 (lines 3,4) nt; or shortened to 24 nt and shuffled (line 6). The numbers in the parenthesis indicate the 3'UTR length. **B**: An alignment for the 3’UTR is shown, wherein the stop codon is underlined, the nucleotides transcribed from the vector are italicized, and the nucleotides from the gene 4 3'UTR are in bold. The alignment was generated manually. The *Renilla* and firefly luciferases activities were measured 6 hours after electroporation. The ratio of *Renilla* to firefly luciferase activities is reported relative to the control (line 1), which was considered 100. The data are the mean ± SEM of three independent experiments in triplicate. A Student’s *t*-test was used (*p<0.05;**p<0,01).

Reporter mRNA with a 5’UTR that is sufficiently long (gene 11; 21 nt) to accommodate conventional scanning-dependent translation initiation or with the short gene 4 5'UTR was combined with the gene 6 or 4 3'UTR and used in our translation assay (Fig 8). A significant difference in translation efficiency was not observed between the four reporters, which eliminates an effect from the presence of a translation enhancer exclusively in the gene 6 3'UTR. If such a signal was present in gene 4 3'UTR, shuffling and/or shortening the gene 4 3'UTR would most likely destroy the signal. Hence, the gene 4 wild type (9 nt-long) 5’UTR was combined with the gene 4 full-length (35-nt) or shortened (20 and 11 nt-long) 3’UTRs (Fig 9). Shortening gene 4 3'UTR to 11 nt reduced translation 4-fold (line 3); but weaker translation was observed with a reporter mRNA bearing the gene 11 5'UTR. This last result indicates that an 11-nt 3’UTR is not sufficiently long to support efficient translation regardless of the 5'UTR. It is highly likely that the distance between the termination codon and 3' end of the RNA bound to NSP3 is not sufficiently large to accommodate a ribosome with a stop codon in the A site; NSP3 would act as a roadblock and render the stop codon inaccessible to the ribosome. Furthermore, combining the same gene 4 5'UTR with a shuffled 35-nt-long 3'UTR did not change the translation efficiency of the reporter (lines 1 and 5). Surprisingly, a shuffled gene 4 3'UTR shortened to 24-nt enhanced translation 2-fold (lines 1 and 6) showing that the viral 3’UTR can be modified to improve translation; changes in the 3’UTR sequence likely modify the mRNA secondary structure and, consequently, the 3' end accessibility to NSP3.

Reducing the 5'UTR to 5-nt did not decrease translation combined with the wild-type 35-nt 3'UTR of gene four (lines 1 and 7). However, translation was slightly weaker when the 5-nt 5'UTR was associated with a shuffled 35-nt 3'UTR (lines 7 and 8).

Thus, whether the reporter mRNA was efficiently translated with the short (9 nt) 5’UTR of gene four was not due to the RNA sequences in the 5' or 3’UTRs.

## Discussion

### I- An improved in vivo assay to study the role of the rotavirus NSP3

We constructed an in vivo assay to study the role of the rotavirus NSP3 and rotavirus mRNA non-coding sequences on rotavirus-like mRNA translation. We improved our [21] or others’ [22, 30] previous experiments in several ways. First, we do not use rotavirus infection, which facilitates an evaluation of NSP3 function independently of changes in cellular physiology induced by virus infection. Second using transient cytoplasmic expression of NSP3 is less laborious and less time-consuming than constitutive expression [23], which facilitates an easy comparison of different wild-type or mutant NSP3 proteins. Third, co-transfection of an RNA standard provides a convenient means to quantify and standardize the results. Fourth, in vitro synthesis of the reporter mRNAs was improved. We used a type IIs restriction enzyme to generate 3’ recessed ends [31] instead of the restriction enzyme KspI, which facilitated synthesis of homogeneous RNAs ending with the desired sequence and eliminate the requirement for recess at the 3' end of the DNA templates [21, 32]. Moreover, using the anti-reverse cap analog (ARCA) produces a higher proportion of correctly capped mRNA molecules [33]. Finally, electroporation facilitates instantaneous delivery of RNA into the cell cytoplasm, whereas lipofection slowly delivers RNA-lipid complexes into the endosomes. Lipid-RNA complexes last a long time in lipofected cells, thus impeding precise RNA quantification [34].

### II- NSP3 stimulates translation of rotavirus-like mRNA in vitro

Using this assay, we show that NSP3 expression enhances rotavirus-like mRNA translation. Capped and uncapped mRNAs translation being stimulated by NSP3 to the same extend (Fig 2), it can be concluded that the NSP3-mediated enhancement of translation is independent of the cap structure.

However, synergy between the cap structure and NSP3 was observed, and the presence of a cap or of NSP3 stimulates translation 5-fold, but the presence of both NSP3 and a cap stimulates translation more than 40-fold. This synergy is comparable to the synergy observed between the cap and PABP on polyadenylated cellular mRNA [35] and renders NSP3 a viral surrogate of PABP. These results are consistent with our in vivo and in vitro translation assays [21, 23] and Chizikoff and Patton’s experiments using infected cells [22], which showed enhanced translation when the reporter mRNA ends with GACC sequence. These results explain why the rotavirus mRNAs transfected into MA104 cells without NSP3 (or NSP3-encoding mRNA) were not translated [36]).

### III- stimulation of translation with low level of NSP3

R-RNA translation is enhanced even with a small quantity of NSP3 barely detectable by a western blot (S2 Fig). This observation could explain why NSP3 depletion only affects viral protein synthesis at early infection times [24] when NSP3 is not abundant. Later during infection, even the low quantities of NSP3 that remain after RNAi might be sufficient to stimulate viral mRNA translation.

Interestingly, slightly but significantly enhanced Nc-RNA GGCC translation was observed in cells expressing NSP3-SA versus cells lacking NSP3 (Fig 3; but not versus cells expressing NSP3-RF). These data suggest that NSP3-SA, similar to NSP3-RRV [23], with which it shares a high amino acids sequence homology [37], exhibits some recognition specificity for this 3' end. Thus, the SA11 strain with its gene 7 non-canonical 3' end (GGCC), which is not well-recognized by NSP3-SA (Fig 4), should produce less NSP3 and may be more sensitive to RNAi directed against NSP3 than other strains. However, whereas cell-culture-adapted rotavirus strains growing in poorly differentiated epithelial cells, such as MA104, may support low levels of NSP3, these non-canonical 3' ends may be detrimental for wild-type rotavirus growth in animals. Similarly, the GAACC and GGCC sequence at the 3' end of gene 5 in SA11 and RRV, respectively, likely greatly impairs production of the anti-interferon protein NSP1 during SA11 or RRV infection [38] because neither NSP3-SA nor NSP3-RRV enhances Nc-RNA translation (Fig 4 and [23]). Less NSP1 would impair subversion of early host antiviral immunity, which would likely impair viral production in wild type mice, as observed with RRV [39].

Using the "T2A" reporters, we show positive feedback between NSP3 and its mRNA. During rotavirus infection, such feedback would facilitate rapid accumulation of the first NSP3 molecules, and once NSP3-dependent translation is triggered, NSP3 is available for other viral mRNA molecules. In the SA11 strain, which does not benefit from the positive feedback for gene 7 mRNA translation (due to gene 7 non-canonical GGCC 3' end), the sustained pace of viral mRNA synthesis might facilitate generation, albeit belatedly, of a sufficient quantity of NSP3.

Interestingly, NSP3-mediated enhanced translation varies depending on the 5' rotavirus UTR (5-fold with the R-RNA with the gene 11 5'UTR used in Figs 3 and 4, 6 to 25-fold with the gene 4 5'UTR used in Fig 7). Using different 5'UTRs might regulate the relative level of different viral proteins to optimize production of virus particles. NSP3-mediated enhanced translation may also depends on the cell line used because much greater translation stimulation was observed with the same reporter mRNA in rotavirus-infected MA104 cells and in cells constitutively expressing NSP3 [23] than in BSRT7 cells. NSP3-mediated enhanced translation may vary depending on the cell physiology; adaptation of the assay described here to MA104 cells used to grow rotavirus may be used to determine whether the cell line plays a role.

### IV- The two domains of NSP3 are together required to stimulate translation

Using mutants of the NSP3 RNA- or eIF4G- binding domain, we show that both domains do not function properly when separated, but they must lie in the same polypeptide to efficiently stimulate translation, which eliminates the hypothesis that NSP3 includes two independent functions [24]. However, partial translation stimulation was observed with the NSP3 RNA-BD and 4G-BD. The 4G BD-dependent translation stimulation may be due to the same general translation stimulation observed in cells expressing full-length NSP3, that we attributed to the enhanced eIF4E-eIF4G interaction due to the NSP3 eIF4G-BD [23]. The mild translation stimulation observed with the NSP3 RNA-BD (3.5-fold) (Fig 4) was not observed using an in vitro assay with recombinant NSP3 and rabbit reticulocyte lysate (RRL) [21]. This stimulation may rather be related to a greater 3'-5' RNA degradation activity in BSR cells than in the RRL system [35]. Thus, RNA-BD stabilization of R-RNA may visibly impact BSR but not RRL nor MA104 translation [23]. However, interestingly, this R-RNA protection from degradation was not detected using an in vivo assay with MA104 cells expressing NSP3, wherein a 100-fold translation stimulation was observed [23]. However, the means for modifying R-RNA expression through other cellular factors interacting with NSP3, such as RoXaN [40, 41], (an A/U-rich RNA-destabilizing element (ARE) RNA-binding protein, [42] the binding domain from which is present on each NSP3 construct used here) remain to be investigated.

### V- Does rotavirus mRNAs translation initiation occur through ribosome scanning?

Our assay facilitated comparison of NSP3- and poly(A)-dependent translation with an emphasis on translation driven by rotavirus gene 4 UTRs. Rotavirus gene 4 is remarkable because it is efficiently translated despite a short 5'UTR. We show that NSP3-dependent translation is less sensitive to 5’UTR length than poly(A)-dependent translation, but the good environment requirement for the translation initiation codon remains. Translation can initiate at a short 5’UTR (<8-nt) without eIF1 [43], but, in this instance and in contrast to our observations, initiation with R-RNAs was observed with a poor Kozak AUG. Thus, R-RNA translation was not likely initiated without eIF1. This weaker requirement for a long 5’UTR neither results from an IRES mode of translation [9] nor from tethering eIF4E, which has been described for histone mRNA [12] because deleting three nucleotides in the middle of the 5'UTR or gene 4 3'UTR deletions or shuffling did not decreased reporter mRNA translation. Translation initiation at a short 5'UTR might proceed through the "RNA looping" mechanism recently described by Paek KY et al., in which translation efficiency depends on the probability of collision between the ribosome and initiation codon [44]. In this translation initiation model, mRNA pseudo circularization by a complex composed of PABP-eIF4G-eIF4E or NSP3-eIF4G-eIF4E " *preferentially facilitates translation from the 5' AUG by the 40S ribosome recruited to the 3' end by shortening the effective distance between the 40S ribosome and the 5' AUG*." [44]. In this instance, the 5' and 3’UTR length influence on translation initiation efficiency is much lower as observed in this study.

Our results using shortened and shuffled 3’UTRs show that the viral 3’UTR can be modified to improve translation; changes in the 3’UTR sequence likely modify the mRNA secondary structure and, consequently, the 3' end accessibility to NSP3. However, a 11-nt 3’UTR strongly decreased reporter translation with the 5’UTR from genes 11 or 4. In this instance, it is highly likely that the distance between the termination codon and 3' end of the RNA bound to NSP3 is not sufficiently large to accommodate a ribosome with a stop codon in the A site; NSP3 would act as a roadblock and render the stop codon inaccessible to the ribosome.

Using our novel in vivo assay, we validated and, more importantly, quantified the roles of NSP3 and regulatory RNA sequences in rotavirus mRNA translation. Our study will be also useful for investigating how other rotavirus proteins or infection-induced physiological changes (e.g., stress and eIF2a phosphorylation) impact rotavirus gene expression.

## Materials and Methods

### Cells

Baby hamster kidney BSRT7 cells [45] were maintained in Eagle’s minimum essential medium (Lonza) supplemented with 10% fetal bovine serum, 100 IU/ml penicillin and 100 μg/mL streptomycin.

### Plasmid constructs and mutagenesis

The deoxy-oligonucleotides used for the plasmid constructs are listed in Table 1.

**Table 1:** The names and DNA sequences of the oligodeoxyribonucleotides used in this study.

OLIGO NAME	polarity	SEQUENCE	USE
F7	**+**	**GATTAGGCGGCCGCTAATACGACTCACTATAGGCTTTTAATGCTTTTCAG**	RT-PCR for cloning RF07 PCR product digested by NotI and AgeI in NotI-XmaI of priboz
R7	**-**	**GATTAGTACCCGGGTCTCCGGTCACATAACGCCCCTATAG**	
BsaupRF07	**+**	**GGCGTTATGTGACCGGAGACCGATGGCATCTCCACCTCCTC**	add BsaI site at the end of the 3'UTR of RF gene 07
BsaloRF07	**-**	**CGAGGAGGTGGAGATGCCATCGGTCTCCGGTCACATAACGCC**	
NcoM4RF07up	**+**	**GGTTGATGCTCACCATGGAGTCTACACAG**	add NcoI site on the fourth codon of NSP3 ORF
NcoM4RF07lo	**-**	**CTGTGTAGACTCCATGGTGAGCATCAACC**	
F11	**+**	**ATAAGAATGCGGCCGCTAATACGACTCACTATAGGCTTTAAAGCGCTACAGTG**	PCR for cloning renilla luciferase ORF between rotavirus UTRs in priboz
R6	**-**	**ATAGTTTAACCGGTCACATCCTCTCACTACG**	
BSAup	**+**	**GTGAGAGGATGTGACCGGAGACCGATGGCATCTCCACCTCCTCG**	add BSA1 site 3' to RF06 UTR (site directed mutagenesis)
BSAlo	**-**	**CGAGGAGGTGGAGATGCCATCGGTCTCCGGTCACATCCTCTCAC**	
NSP3stopup	**+**	**GTCTACACAGCAGTAGGCTAGTTCG**	introduce a stop codon at amino acid postion 7 of NSP3ORF (site directed mutagenesis)
NSP3stoplo	**-**	**CGAACTAGCCTACTGCTGTGTAGAC**	
NSP3 mutRNAup	**+**	**ATTTGGTTCAGCAGCAGCTGCTAGAAATTGGATGGCTGA**	change positions 83-84(RN) of the RNA-binding domain of NSP3 into alanines (site directed mutagenesis)
NSP3mutRNAlo	**-**	**TCAGCCATCCAATTTCTAGCAGCTGCTGCTGAACCAAAT**	
NSP3mutd4Gup	**+**	**GAATGGTACCTGAAATGATAGCAATTGCCTGATG**	to introduce a stop codon at position 251 of NSP3; deletion of the eIF4G binding domain (site directed mutagenesis)
NSP3mutd4Glo	**-**	**CATCAGGCAATTGCTATCATTTCAGGTACCATTC**	
NSP3stopup	**+**	**GTCTACACAGCAGTAGGCTAGTTCGATTATTAAC**	to introduce a stop codon at position 6 of NSP3
NSP3stoplo	**-**	**GTTAATAATCGAACTAGCCTACTGCTGTGTAGAC**	
DIR-SARR07	**+**	**GATTAGGCGGCCGCTAATACGACTCACTATAGGCTTTTAATGCTTTTCAG**	RT-PCR of SA11 gene 07. PCR product digested by NotI and XmaI and cloned in priboz
REV-SARR07	**-**	**GATTAGTACCCGGGTCTCCGGACACATAACGCCCCTATAG**	
NonaGAACC/Bsa up	**+**	**GTGAGAGGATGTGAACCGGAGACCGATGGCATCTCCACCTCCTCG**	to mutate the 3' GACC sequence in GAACC (site directed mutagenesis)
NonaGAACC/Bsa lo	**-**	**CGAGGAGGTGGAGATGCCATCGGTCTCCGGTTCACATCCTCTCAC**	
NonaGGCC/Bsa up	**+**	**GTGAGAGGATGTGGCCGGAGACCGATGGCATCTCCACCTCCTCG**	to mutate the 3' GACC sequence in GGCC (site directed mutagenesis)
NonaGGCC/Bsa lo	**-**	**CGAGGAGGTGGAGATGCCATCGGTCTCCGGCCACATCCTCTCAC**	
oligodTBSAEcoUp	**-**	**CCGGTGCGCAGCTGATCATGGAATTCGGTCTCTTTTTTTTTTTTTTTTT**	reverse transcription of polyadenylated dsRNA
BSAEcoUAP	**-**	**CCGGTGCGCAGCTGATCATGGAATTC**	amplification of polyadenylated cDNA
BAMRF6UTR3	**+**	**ATGATCGGATCCTGAGGACCAAGCTAACCACTTGG**	amplification of polyadenylated RF06 3'UTR cDNA
GFPncoup	**+**	**GTGAGCCATGGTGAGCAAGGGCGAGGAG**	amplification of eGFP ORFby PCR with a NcoI site at both ends
GFPncolo	**-**	**GACGAGCTGTACAAGTccatggCGACTTT**	
GFPT2ANSP3UP	**+**	**GTACAAGGAAGGCCGGGGAAGCCTGCTGACCTGCGGGGACGTGGAGGAAAACCCCGGACCAATGGAGT**	insertion of the T2A coding sequence between BsrGI and AccI sites
GFPT2ANSP3LO	**-**	**AGACTCCATTGGTCCGGGGTTTTCCTCCACGTCCCCGCAGGTCAGCAGGCTTCCCCGGCCTTCCTT**	
5’ UTR 04RF-9up	**+**	**GGCCGCTAATACGACTCACTATAGGCTATAAAATGGCTT**	cloning gene four (9 nucleotide-long) 5'UTR upstream Renilla luciferase
5’ UTR 04RF-9lo	**-**	**CGAAGCCATTTTATAGCCTATAGTGAGTCGTATTAGC**	
5’ UTR 04 RF-5up	**+**	**GGCCGCTAATACGACTCACTATAGGCAAATGGCTT**	cloning 5 nucleotide-long 5'UTR upstream Renilla luciferase
5’ UTR 04 RF-5lo	**-**	**CGAAGCCATTTGCCTATAGTGAGTCGTATTAGC**	
5’ UTR RF-2up	**+**	**GGCCGCTAATACGACTCACTATAGGATGGCTT**	cloning 2 nucleotide-long 5'UTR upstream Renilla luciferase
5’ UTR RF-2lo	**-**	**CGAAGCCATCCTATAGTGAGTCGTATTAGC**	
AUG Stop 04RF-9up	**+**	**GGCCGCTAATACGACTCACTATAGGCTATAAATAAGCTT**	cloning gene four 5'UTR with stop in place of start codon upstream Renilla luciferase
AUG Stop 04RF-9lo	**-**	**CGAAGCTTATTTATAGCCTATAGTGAGTCGTATTAGC**	
no kozak +4-9up	**+**	**GGCCGCTAATACGACTCACTATAGGCTATAAAATGTCTT**	cloning gene four 5'UTR with a modified "Kozak" (position +4) upstream Renilla luciferase
no kozak+4-9lo	**-**	**CGAAGACATTTTATAGCCTATAGTGAGTCGTATTAGC**	
no kozak-3+4-9up	**+**	**GGCCGCTAATACGACTCACTATAGGCTATCAAATGTCTT**	cloning gene four 5'UTR 9 nucleotide long underlined) with a modified "Kozak" (positions +4 and -3, bold) upstream Renilla luciferase
no kozak-3+4-9lo	**-**	**CGAAGACATTTGATAGCCTATAGTGAGTCGTATTAGC**	
no kozak -3+4-5up	**+**	**GGCCGCTAATACGACTCACTATAGGCAAATGTCTT**	cloning gene four 5 nucleotide long 5'UTR with a modified "Kozak" (position -3 and +4) upstream Renilla luciferase
no kozak-3+4-5lo	**-**	**CGAAGACATTTGCCTATAGTGAGTCGTATTAGC**	
no kozak-3-5up	**+**	**GGCCGCTAATACGACTCACTATAGGCAAATGGCTT**	cloning gene four 5 nucleotide long 5'UTR with a modified "Kozak" (position +4)upstream Renilla luciferase
no kozak-3-5up	**-**	**CGAAGCCATTTGCCTATAGTGAGTCGTATTAGC**	
3’ UTR 04 RF-35up	**+**	**GATCCGCAACTGACAGAGGATGTGACCGGAGACCG**	cloning gene four 3'UTR downstream Renilla luciferase
3’ UTR 04 RF-35lo	**-**	**AATTCGGTCTCCGGTCACATCCTCTGTCAGTTGCG**	
3’ UTR shuffled-35up	**+**	**GATCCCTGCGGAAAAATGGACTGGACCGGAGACCG**	cloning gene four shuffled 3'UTR downstream Renilla luciferase
3’ UTR shuffled-35lo	**-**	**AATTCGGTCTCCGGTCCAGTCCATTTTTCCGCAGG**	
3’ UTR 04 RF-11up	**+**	**CTAGTGACCGGAGACCG**	cloning gene four short(11 nucleotides) 3'UTR downstream Renilla luciferase
3’ UTR 04 RF-11lo	**-**	**AATTCGGTCTCCGGTCA**	
3’ UTR 04RF-20up	**+**	**CTAGCAGAGGATGTGACCGGAGACCG**	cloning gene four short (20 nucleotides) 3'UTR downstream Renilla luciferase
3’ UTR 04RF-20lo	**-**	**AATTCGGTCTCCGGTCACATCCTCTG**	
3’ UTR shuffled-24up	**+**	**CTAGCTAGCAGAAAATGTGACCGGAGACCG**	cloning shuffled (24) 3'UTR downstream Renilla luciferase
3’ UTR shuffled-24lo	**-**	**AATTCGGTCTCCGGTCACATTTTCTGCTAG**	
FlucACC65up	**+**	**GCGGAAAGATCGCCGTAGGGTACCTTCTAGAGTCGGGG**	to introduce an Acc65I site in Fluc ORF of pGL3
FlucACC65lo	**-**	**CCCCGACTCTAGAAGGTACCCTACGGCGATCTTTCCGC**	

### NSP3, eGFP and control expression vectors

To construct the NSP3-RF expression vectors (Fig 1A), viral dsRNA was extracted from the culture medium as described in [46]. Further, gene 7 was reverse transcribed with the direct primer (F7) bearing a NotI restriction site followed by the T7 promoter fused to the first 20 nt of the RF gene 7 5’UTR and a reverse primer (R7) bearing a sequence complementary to the last 20 nt of gene 7 followed by an AgeI restriction site (Table 1). The PCR fragments were treated with NotI and AgeI and cloned into the NotI- and XmaI-linearized pRiboz vector [47], which produced the plasmid pRiboz RF07. Next, a BsaI restriction site was introduced at the 3’ end of the 3’UTR (Fig 1A) through site-directed mutagenesis using the oligonucleotides BsaupRF07 and BsaloRF07 (Table 1). Note that introduction of the BsaI site disabled the ribozyme pRiboz. A NcoI site was added to the second AUG (M4) of NSP3RF ORF using the oligonucleotides NcoM4RF07up and NcoM4RF07lo (Table 1), which produced pribozRF07Nco/Bsa. The NotI-NcoI fragment from pribozRF07Nco/Bsa was then replaced by a NotI-NcoI fragment from a modified pTM1 plasmid [48], [49] bearing a NotI site upstream of a T7 promoter followed by the EMCV internal ribosome entry site (IRES), which produced the plasmid pT7-ires-NSP3 (Fig 1A). The pT7-ires-NSP3SA vector encoding NSP3 from the SA11 RVA strain was generated through RT-PCR using dsRNA extracted from SA11 virus preparation as the template and the oligonucleotides DIR-SARR07 and REV-SARR07 (Table 1) as primers. The PCR product was then digested by NotI and XmaI and cloned into priboz. The T7promoter and 5'UTR were exchanged for the T7promoter and EMCV IRES purified from pT7-ires-NSP3 as a NotI-AccI fragment, which produced the pT7-ires-NSP3-SA expression vector.

Mutations were introduced in the NSP3-RF RNA- and eIF4G-binding domains through site-directed mutagenesis. The amino acids 83(R) and 84(N) in contact with the RNA [17] were mutated to alanines using the oligonucleotides NSP3mutRNAup and NSP3mutRNAlo (Table 1) to generate the pT7-Ires-NSP3dRN used to express the NSP3 eIF4G binding domain (4G-BD). A stop codon was introduced at position 251 (using the oligonucleotides NSP3mutd4Gup and NSP3mutd4Glo (Table 1)), which deleted the NSP3 4G-BD in pT7-ires-NSP3d4G [19, 50] used to express the NSP3 RNA-binding domain (RN-BD).

Two negative controls were used; pT7-Ires-eGFP expressed the protein eGFP instead of NSP3, and pT7-ires-NSP3-KO encoded only the first 6 amino acids of NSP3-RF. To generate pT7-Ires-eGFP, the eGFP coding sequence was retrieved from the peGFPN1 (Clontech) as an NcoI-XbaI fragment and cloned into pT7-RF06-Fluc-RF06 [21]. PCR amplification using the oligonucleotides F11 and R6 (Table 1) and cloning into the pribozdelta NotI and XmaI sites [49] produced pribozdelta eGFP. The 5’UTR was then exchanged for the EMCV IRES from the modified pTM1 (see above) to produce pT7-ires-eGFP. To generate pT7-ires-NSP3-KO, the 7th codon of the NSP3 ORF was mutated into a stop codon through site-directed mutagenesis using the oligonucleotides NSP3stopup and NSP3stoplo (Table 1).

### Plasmids for in vitro transcription of the reporter and standard RNAs

A schematic representation of the plasmids used to synthesize the reporter mRNAs is presented in S1 Fig. The plasmid pT7-RF-Rluc-Bsa-GACC was used for in vitro synthesis of rotavirus-like mRNAs ending with a canonical GACC 3' end (R-RNA). To produce the pT7-RF-Rluc-Bsa-GACC, the firefly luciferase ORF in pT7-RF06-FLuc-RF06 [21] was amplified using PCR with the oligonucleotides F11 and R6 (Table 1) and then cloned into the pRiboz NotI and XmaI sites [47]. A BsaI site was then introduced through site-directed mutagenesis downstream of the 3’UTR (oligonucleotides BSAup and BSAlo (Table 1)). The Fluc ORF was then mutated into the *Renilla* luciferase ORF, which was obtained from pGL3basic (Promega) as an NcoI-XbaI fragment.

Site-directed mutagenesis (Quick-change, Stratagene) using the oligonucleotide pairs NonaGAACC/Bsa and NonaGGCC/Bsa (up and lo, Table 1) on pT7-RF-Rluc-GACC-Bsa produced pT7-RF-Rluc-GGCC-Bsa and pT7-RF-Rluc-GAACC-Bsa. The plasmids were used for in vitro synthesis of the rotavirus-like mRNAs ending with non-canonical 3' ends Nc-RNA-GGCC and Nc-RNA-GAACC, respectively.

To produce a reporter mRNA (pA-RNA) that differs from the R-RNA reporter by only a stretch of adenines, genomic dsRNA purified from RF RVA virions was polyadenylated with *E*. *coli* poly(A)-polymerase and ATP for 20 min and reverse transcribed with an oligo-dT primer (oligodTBSAEcoUp; Table 1). The polyadenylated 3’UTR from gene 6 was then amplified by PCR using the oligonucleotides BSAEcoUAP and BAMRF6UTR3 (Table 1). The PCR products were separated by electrophoresis on an agarose gel, and the PCR products > 250 bp were purified and cloned into pGEMTeasy following the T/A cloning protocol provided by the supplier (Promega). Several clones were sequenced, and a clone with 66 adenines was selected. The pT7-RF-RLuc-Bsa-GACC BamHI-EcoRI fragment containing the RF06-3'UTR was substituted by the BamHI-EcoRI fragment containing the polyadenylated RF06-3'UTR to produce pT7-RF-Rluc-p(A)-Bsa (S1 Fig).

The 5’UTR and initiation codon environment in pT7-RF-Rluc-GACC-Bsa and pT7-RF-Rluc-p(A)-Bsa were modified by cloning pairs of complementary oligonucleotides (5'UTRgene04RF-9up to nokozak-3-5, Table 1) between the NotI and BstBI sites of the pT7-RF-Rluc vectors.

The pT7-RF-Rluc-GACC-Bsa 3’UTR was modified by cloning two complementary, annealed oligonucleotides between the BamHI and EcoRI sites (oligonucleotides 3'UTR 04RF-35, shuffled-35) or the XbaI and EcoRI sites (oligonucleotides 3'UTR 04RF-11, 04RF-20 and shuffled-24) of pT7-RF-Rluc-GACC-Bsa.

The standard EMCV Fluc RNA was generated through in vitro transcription of the pEMCV-Fluc plasmid linearized through treatment with the restriction enzyme EcoRI. To produce the pEMCV-Fluc plasmid, the pT7-RF06-Fluc-RF06 Fluc ORF [21] was amplified by PCR using oligonucleotides F11 and R6 (Table 1) and cloned between the pRibozdelta NotI and XmaI sites [49]. Replacing RF 5’UTR with the EMCV IRES as a NotI-NcoI fragment was performed as described above for the NSP3 expression vectors.

The PCR fragments and annealed oligonucleotides were fully sequenced after cloning into the target plasmids. For site-directed mutagenesis, the entire functional (from the T7 promoter to the T7 terminator) unit was sequenced. All plasmid constructs were validated through restriction enzyme mapping.

### Plasmids for in vitro transcription of the Firefly-T2A-NSP3 mRNA

The eGFP ORF was first introduced in frame with the NSP3 ORF into pribozRF07Nco/Bsa (see above) as an NcoI PCR fragment (using GFPncoup and GFPncolo as primers, Table 1). A T2A sequence [26] was then introduced as two complementary oligonucleotides (oligonucleotides GFPT2ANSP3up/lo) between the BsrGI and AccI sites. The eGFP ORF was then replaced by the firefly luciferase ORF as an NcoI-Acc65 fragment (generated after site-directed mutagenesis of the firefly ORF in pGL3 using the oligonucleotides Acc65up and Acc65lo (Table 1)) between the NcoI and BsrGI sites. The eIF4G1- and RNA-binding sites and 3'UTR were mutated as described above.

### In vitro transcription

The capped RNA molecules were generated through in vitro transcription of the template plasmids linearized by BsaI using the mMessage mMachine T7ultra kit (Ambion). The DNA was removed through an enzymatic treatment (15 min 37°C) with RNAse-free DNase, and the RNA molecules were purified using MegaClear (Ambion) silica spin columns to eliminate unincorporated cap analogues and nucleotides prior to ethanol precipitation. The purified RNA molecules were quantified using a spectrophotometer (Nanodrop), then controlled using denaturing agarose gel electrophoresis and stored in aliquots at minus 80°C. Uncapped mRNA or IRES mRNA molecules were synthesized using the same linearized templates and T7mega script kit (Ambion), which does not include the ARCA cap analog [51].

Unless stated otherwise, the reporter mRNA was capped. The reporter mRNA is abbreviated as R-RNA (cap-Rluc-GACC mRNA) for rotavirus-like reporter mRNA, Nc-RNA for rotavirus-like reporter mRNA ending with the non-canonical 3' end GAACC or GGCC and pA-RNA (cap-Rluc-p(A)) for poly(A) mRNA.

### Cell transfection, electroporation and luminescence measurements

The DNA was introduced into BSRT7 by lipofection using Lipofectamine 2000 reagent in accordance with the supplier’s instructions (Life Technologies). Twenty-four hours later, RNA was introduced into BSRT7 cells using a Neon electroporation device (Life Technologies). The cells (10^6^) were suspended in R buffer (Life Technologies) with 50 ng of reporter mRNA (and 1 μg of standard RNA). The conditions for optimal electroporation were determined for the BSRT7 cells as two 20 ms pulses of 1400 V. After electroporation, complete culture medium was immediately added, and the cells were incubated at 37°C.

At the indicated times, the cells were lysed in the passive lysis buffer (Promega) for 15 min and then frozen at -20°C. Luminescence was measured using the Dual-Luciferase-Reporter Assay System (Promega) and a luminometer (Sirius, Berthold). The results are from three experiments conducted in triplicate with three different reporter mRNA preparations. The results were analyzed using Student's two-tailed *t*-test.

### Western-blot

Proteins separated using SDS-PAGE were transferred to low-fluorescence PVDF membranes and probed with a rabbit polyclonal antibody against NSP3 (4–150) **[41]** and antiGAPDH monoclonal antibody (Abcam-ab8245) at 1/1000 and 1/40000 dilutions, respectively. Anti-rabbit and anti-mouse secondary antibodies coupled to Dyelight 800 (Perbio) or IRdye 700 (LI-COR) were used at a 1/10000 dilution. Western blots were visualized using an OdysseyFC imager and quantified using Image Studio software (LI-COR).

## Supporting Information

S1 Fig Schematic representations of the NSP3 expression, reporter and standard plasmid vectors used in this study. A: Schematic representation of the NSP3 expression vector, pT7-ires-NSP3. B: Schematic representations of 1- the vector pT7 RF-Rluc-GACC-Bsa used for in vitro synthesis of the rotavirus-like (R-RNA) reporter mRNA and 2- the vector pT7RF-Rluc-p(A)-Bsa used for in vitro synthesis of the polyadenylated RNA reporter (pA-RNA). The DNA sequences (top and bottom strands) at the start and end of the T7 RNA polymerase transcription unit are indicated. The start site (+1) is indicated, the DNA sequence recognized by the BsaI restriction enzyme is underlined, and the slashes indicate the position of the cuts. The rotavirus 3' consensus sequence (GACC) mutated in the Nc-RNAs vectors is indicated in bold. The 5' and 3' end sequences for the RNA produced by the T7 RNA polymerase (that uses the bottom DNA strand as a template) with the plasmids cut by BsaI are indicated. C: Schematic representation of the vector pT7-ires-Fluc used for in vitro synthesis of the transfection standard RNA (S-RNA). The positions of the restriction sites used for plasmid constructions (see text) are also indicated. pT7: T7 RNA polymerase promoter; terT7: T7 RNA polymerase terminator; IRES: EMCV Internal Ribosome Entry Site; Rluc: *Renilla* luciferase ORF; FLuc: firefly (*Photinus pyralis*) luciferase ORF; NSP3: NSP3 ORF.
(TIFF)

S2 Fig An in vivo assay for NSP3 function in translation. **A:** Cytoplasmic expression of NSP3 in BSRT7 cells. Lysates from BSRT7 cells transfected for 24 h with the indicated quantities (μg) of pT7Ires RF07 were analyzed by western blot with an anti-NSP3 rabbit polyclonal antibody and a mouse monoclonal antibody against cellular protein GAPDH (used as a loading control). The ratio of NSP3 versus GAPDH fluorescence (NSP3/GAPDH) is indicated at the bottom of the figure. **B:** The NSP3/GAPDH fluorescence ratio (i.e., NSP3 quantity) is presented as a function of the quantity of plasmid pT7-Ires-NSP3 transfected. **C:** Reporter R-RNA translation as a function of the time after electroporation; capped reporter mRNA (R-RNA) was electroporated into BSRT7 cells expressing NSP3, and the *Renilla* luciferase activity (arbitrary units) was measured at different times after electroporation. **D:** Reporter R-RNA translation as a function of NSP3 expression; capped R-RNA was electroporated in BSRT7 cells expressing increasing quantities of NSP3, and the *Renilla* luciferase activity (arbitrary units) was measured 6 hours after electroporation. **E:** Translation of the standard mRNA is not affected by NSP3 expression. The standard mRNA (ires-Fluc) was transfected into cells expressing NSP3 or eGFP(-), and the firefly luciferase activity (arbitrary units) was measured 6 hours after electroporation. The data are the mean ± standard error of the mean (SEM) for three independent experiments in triplicate.
(TIFF)

S1 Text Detailed description of the translation assay. (DOCX)
