# Supplementary figures and images for: Challenging the Roles of NSP3 and Untranslated Regions in Rotavirus mRNA Translation
Source: PLoS One. 2016 Jan 4;11(1):e0145998. doi: 10.1371/journal.pone.0145998 (PMC4699793; doi:10.1371/journal.pone.0145998)

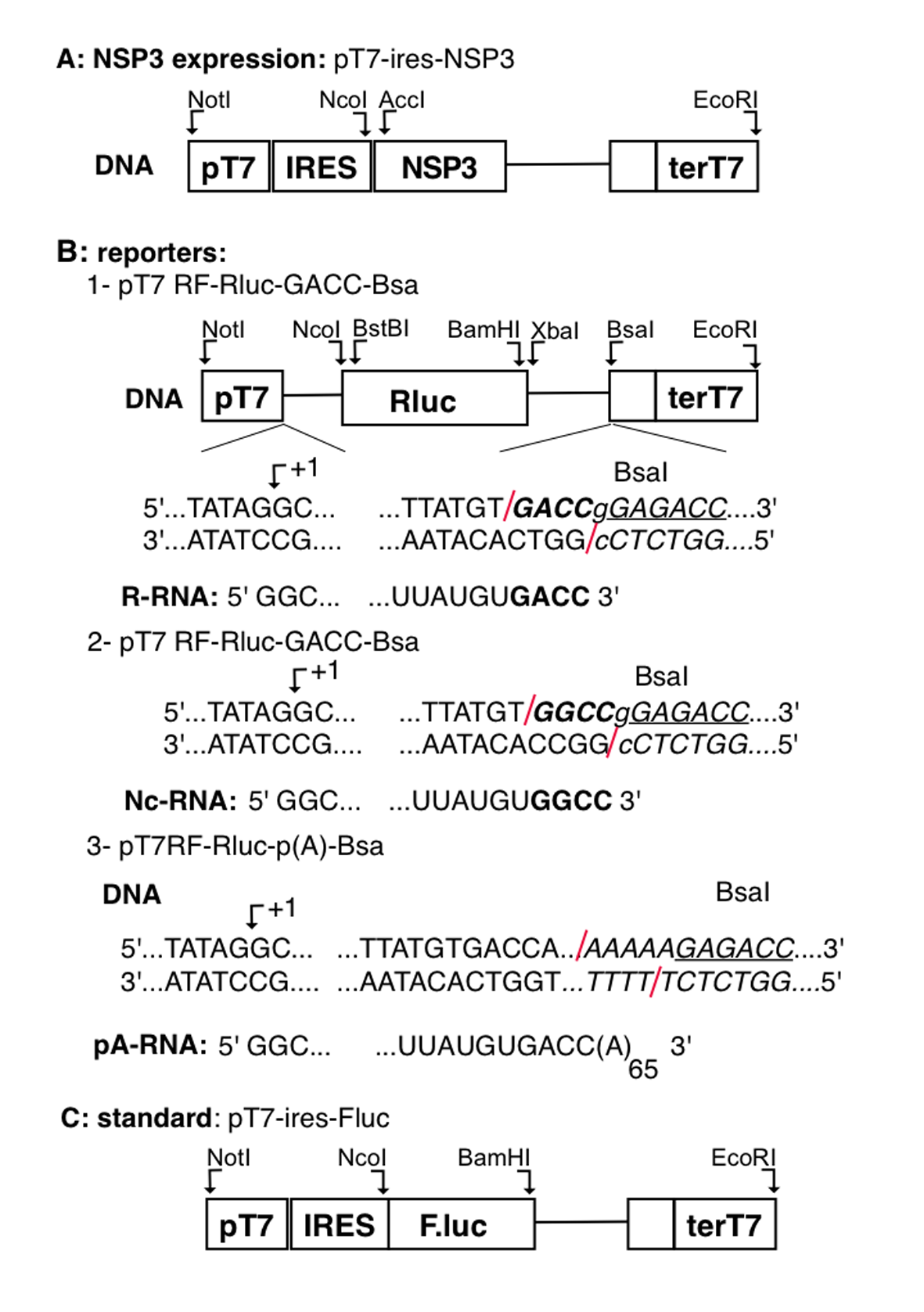

Supplement: S1 Fig — A: Schematic representation of the NSP3 expression vector, pT7-ires-NSP3. B: Schematic representations of 1- the vector pT7 RF-Rluc-GACC-Bsa used for in vitro synthesis of the rotavirus-like (R-RNA) reporter mRNA and 2- the vector pT7RF-Rluc-p(A)-Bsa used for in vitro synthesis of the polyadenylated RNA reporter (pA-RNA). The DNA sequences (top and bottom strands) at the start and end of the T7 RNA polymerase transcription unit are indicated. The start site (+1) is indicated, the DNA sequence recognized by the BsaI restriction enzyme is underlined, and the slashes indicate the position of the cuts. The rotavirus 3' consensus sequence (GACC) mutated in the Nc-RNAs vectors is indicated in bold. The 5' and 3' end sequences for the RNA produced by the T7 RNA polymerase (that uses the bottom DNA strand as a template) with the plasmids cut by BsaI are indicated. C: Schematic representation of the vector pT7-ires-Fluc used for in vitro synthesis of the transfection standard RNA (S-RNA). The positions of the restriction sites used for plasmid constructions (see text) are also indicated. pT7: T7 RNA polymerase promoter; terT7: T7 RNA polymerase terminator; IRES: EMCV Internal Ribosome Entry Site; Rluc: Renilla luciferase ORF; FLuc: firefly (Photinus pyralis) luciferase ORF; NSP3: NSP3 ORF. (TIFF) [file pone.0145998.s001.tiff]

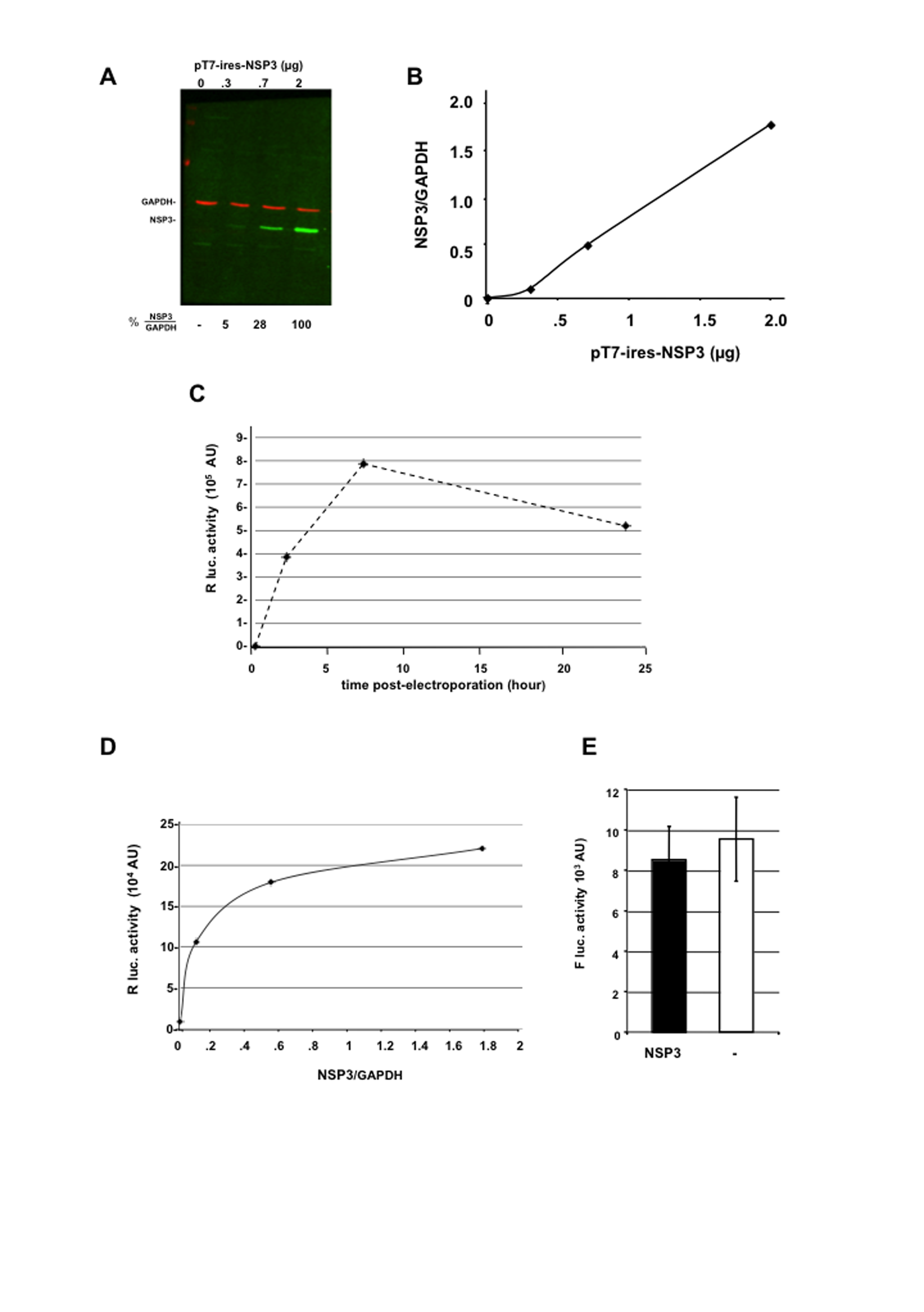

Supplement: S2 Fig — A: Cytoplasmic expression of NSP3 in BSRT7 cells. Lysates from BSRT7 cells transfected for 24 h with the indicated quantities (μg) of pT7Ires RF07 were analyzed by western blot with an anti-NSP3 rabbit polyclonal antibody and a mouse monoclonal antibody against cellular protein GAPDH (used as a loading control). The ratio of NSP3 versus GAPDH fluorescence (NSP3/GAPDH) is indicated at the bottom of the figure. B: The NSP3/GAPDH fluorescence ratio (i.e., NSP3 quantity) is presented as a function of the quantity of plasmid pT7-Ires-NSP3 transfected. C: Reporter R-RNA translation as a function of the time after electroporation; capped reporter mRNA (R-RNA) was electroporated into BSRT7 cells expressing NSP3, and the Renilla luciferase activity (arbitrary units) was measured at different times after electroporation. D: Reporter R-RNA translation as a function of NSP3 expression; capped R-RNA was electroporated in BSRT7 cells expressing increasing quantities of NSP3, and the Renilla luciferase activity (arbitrary units) was measured 6 hours after electroporation. E: Translation of the standard mRNA is not affected by NSP3 expression. The standard mRNA (ires-Fluc) was transfected into cells expressing NSP3 or eGFP(-), and the firefly luciferase activity (arbitrary units) was measured 6 hours after electroporation. The data are the mean ± standard error of the mean (SEM) for three independent experiments in triplicate. (TIFF) [file pone.0145998.s002.tiff]
