# Supplementary material for: Challenging the Roles of NSP3 and Untranslated Regions in Rotavirus mRNA Translation
Source: PLoS One. 2016 Jan 4;11(1):e0145998. doi: 10.1371/journal.pone.0145998 (PMC4699793; doi:10.1371/journal.pone.0145998)
Supplement: S1 Text — (DOCX) [file pone.0145998.s003.docx]

**Supporting information**

**A quantitative in vivo assay to study the role of NSP3 and RNA sequences on the translation efficiency of rotavirus-like mRNA.**

**I - Expression of NSP3.**

In our hands, transient NSP3 expression using expression vectors based on nuclear transcription was not efficient*.* Thus, we used cytoplasmic gene expression under control of the T7 promoter and EMCV IRES using BSRT7 cells, which is a BHK derivative constitutively expressing the T7 RNA polymerase [1]. A schematic representation of the expression vector bearing the NSP3 ORF is presented in S1A fig. When the BSRT7 cells were transfected with an NSP3 expression vector, expression of the viral protein was detected by western-blot 24 hours after lipofection and increased almost linearly with increasing quantities of plasmid (S2 A and B fig.).

**II- Reporter mRNA.**

Schematic representations of the plasmids used to synthesize the reporter mRNA, R-RNA and pA-RNA are shown in Figure S1B. The DNA template (pT7-RF-Rluc-GACC-Bsa) is made of (from 5’ to 3’); the T7 promoter fused to the 5’ non coding sequence of rotavirus RF gene 11, the *Renilla* luciferase coding sequence, and the 3’ non coding sequence of rotavirus RF gene 6 followed by a BsaI restriction site. BsaI is a type IIs restriction enzyme that recognizes an asymmetric DNA sequence (GAGACC) and cleaves both DNA strands at fixed positions several base pairs away from the recognition site. The BsaI restriction site is positioned such that the DNA strand used as a template by the T7 RNA polymerase ends with CTGG (TTTT for pT7-RF-Rluc-p(A)). Thus, run-off transcription of a BsaI-linearized plasmid by the T7 RNA polymerase produces RNA with the GACC rotavirus consensus sequence at the 3’ end (or 65 A for pT7-RF-Rluc-p(A)). Mutations at the 3’ end can be easily introduced through site-directed mutagenesis, which was performed for synthesis of rotavirus-like mRNA ending with a non-canonical 3' sequence (Nc-RNA GAACC and Nc-RNA GGCC; see below). Further, the full 5’ or 3’UTRs can be modified by cloning annealed oligonucleotides with cohesive ends.

We introduced RNA into BSRT7 cells using electroporation, not lipofection, because it instantaneously delivers mRNA into the cell cytoplasm, whereas mRNA release from lipid complexes is slow and generates prolonged expression [2]. Further, in contrast to lipofection, electroporation does not interfere with the RNA half-life [3].

To determine the optimal time for cell recovery after electroporation, the cells transfected with 2 μg of the NSP3 expression vector pT7-ires-RF07 for 24 h, were electroporated with R-RNA and recovered 1, 6 or 24 hours after electroporation. Figure S2C shows that the maximum expression of *Renilla* luciferase was observed approximately 6 hours after electroporation; this time frame was used for subsequent experiments.

When BSRT7 cells expressing increasing quantities of NSP3-RF were electroporated with R-RNA, the *Renilla* activities increased steadily with the level of NSP3 (Figure S2D). In cells transfected with the highest (2 μg) level of plasmid, *Renilla* luciferase expression increased 20-fold relative to cells that did not express NSP3. Notably, even minute quantities of NSP3 that are only slightly visible on a western blot (second lane Figure S2A) enhanced R-RNA translation.

**Ic- Choice of a standard RNA for electroporation**

To quantitatively compare different NSP3 proteins or reporter RNAs mutants, an RNA transfection standard was constructed. RNA quantification using RT-qPCR is an expensive and tedious means of normalizing reporter expression, and we have shown that NSP3 expression does not stabilize R-RNA [4]. To standardize Rluc reporter expression, we investigated mRNA molecules that encode firefly luciferase (Fluc), the expression for which would not be impaired by NSP3 expression. Translation under viral IRES control occurs independently of several translation factors [5, 6]; thus, translation of transfected Fluc mRNAs (non-polyadenylated) under control of the cricket paralysis virus (CrPV), encephalomyocarditis virus (EMCV) or hepatitis C virus (HCV) IRES were examined in BSRT7 cells expressing NSP3. The mRNA with CrPV or HCV IRES required transfection of high levels of RNA for an optimal Fluc signal; however, transfection of mRNA with the EMCV IRES yielded a good Fluc expression with a moderate (1 μg/10^6^ cells) quantities of mRNA, which would not compete with the reporter mRNA. Furthermore, when the EMCV standard RNA was electroporated with R-RNA, its expression was not significantly modified by NSP3 expression (Figure S2E). Thus, the EMCV-FLuc mRNA was used as an electroporation standard in subsequent experiments. The *Renilla* activity was reported relative to the firefly activity (R/F), and, to facilitate comparisons, the R/F ratio obtained using R-RNA electroporated into NSP3-expressing cells, was considered 100.

**References**

1. Buchholz UJ, Finke S, Conzelmann KK. Generation of bovine respiratory syncytial virus (BRSV) from cDNA: BRSV NS2 is not essential for virus replication in tissue culture, and the human RSV leader region acts as a functional BRSV genome promoter. J Virol. 1999;73(1):251-9.

2. Tavernier G, Andries O, Demeester J, Sanders NN, De Smedt SC, Rejman J. mRNA as gene therapeutic: how to control protein expression. Journal of controlled release : official journal of the Controlled Release Society. 2011;150(3):238-47.

3. Barreau C, Dutertre S, Paillard L, Osborne HB. Liposome-mediated RNA transfection should be used with caution. RNA. 2006;12(10):1790-3.

4. Gratia M, Sarot E, P. V, Charpilienne A, Baron CH, Duarte M, et al. Rotavirus NSP3 is a translational surrogate of the PABP-poly(A) complex. J Virol. 2015 Sep;89(17):8773-82.

5. Balvay L, Soto Rifo R, Ricci EP, Decimo D, Ohlmann T. Structural and functional diversity of viral IRESes. Biochim Biophys Acta. 2009;1789(9-10):542-57.

6. Bushell M, Sarnow P. Hijacking the translation apparatus by RNA viruses. J. Cell Biol. 2002;158(3):395-9.
